# Bridging Electron Density Relaxation and XPS Spectroscopy: Orbital-Resolved Trends in Core-Ionized Au_*m*_ (*m* = 4, 10, 20) and (TiO_2_)_*n*_ (*n* = 2–4) Clusters

**DOI:** 10.1021/acs.jpca.6c01107

**Published:** 2026-08-28

**Authors:** Bartłomiej Gostyński

**Affiliations:** Department of Biophysics, Institute of Materials Science and Engineering, ul. Stefanowskiego 1/15, 90-924 Łódź, Poland; Centre of Molecular and Macromolecular Studies, Polish Academy of Sciences, ul. Sienkiewicza 112, 91-363 Łódź, Poland

## Abstract

Core electron binding energies (CEBEs) in nanomaterials encode element-specific chemical information, yet their interpretation is often complicated by quantum size effects and site-to-site heterogeneity in small finite systems. Here, I use all-electron Δ-Self Consistent Field (ΔSCF) density functional theory to quantify site-resolved CEBEs and the accompanying orbital-level relaxation response in two chemically distinct cluster families: metallic Au_*m*_ (*m* = 4, 10, 20) and anatase-like (TiO_2_)_*n*_ (*n* = 2–4), including multiple structural isomers. Beyond total-energy differences, I analyze the orbital-resolved Kohn–Sham eigenvalue shift, Δε, induced by a localized core–hole. Finally, I assess a summed eigenvalue-shift descriptor, ΣΔε, as a compact reduction of these data. The results highlight the intrinsically quantum-mechanical origin of core-level shifts in finite systems and suggest that orbital-resolved relaxation patterns may serve as auxiliary, physically interpretable descriptors in future core-level spectroscopy studies.

## Graphical abstract

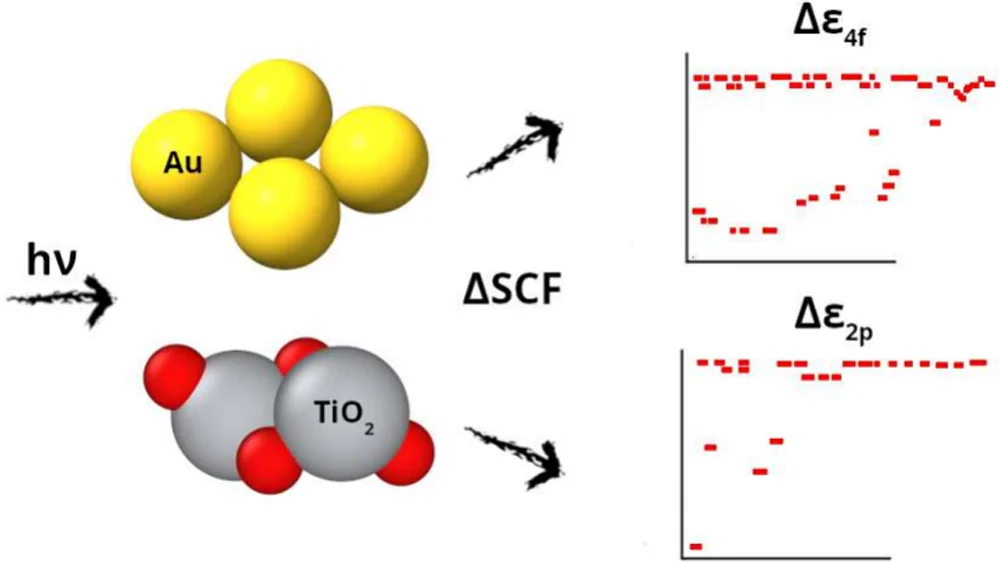

## 1 Introduction

X-ray photoelectron spectroscopy (XPS) has long been an important tool for the characterization of nanomaterials. However, in finite nanoscale systems, the interpretation of core electron binding energies (CEBEs) is often more complex than that in bulk materials due to their structural heterogeneity, the coexistence of chemically or coordinatively distinct atomic sites, and quantum size effects. Therefore, a need for theory appeared that could overcome these experimental difficulties and enable a more unequivocal interpretation of the data.

Theoretical modeling of CEBEs is usually performed via the self-consistent or perturbative frameworks, both frequently based on density functional theory (DFT). The first branch is implemented via the Δ-Self Consistent Field (ΔSCF) approach, whereas the second one is based on response theory via Green’s functions (GW). In the former, the core–electron binding energy is calculated as the total energy difference between the ground state and the final state with a core–hole,^1^ and the latter expresses the core–hole response of the system as a perturbation, avoiding an explicit optimization of the wave function of a core-ionized system.^2^ Therefore, the GW method is theoretically regarded as an improvement upon the limitations of traditional Δ approaches, albeit GW methods are known to have a large starting point dependence on the parent DFT functional or the accuracy of predicting CEBEs for more sophisticated GW approaches cannot be guaranteed to be improved.^2,3^ Moreover, the substantial computational cost renders their usage for prediction of XPS spectra for larger systems impractical.^1^

In addition to this, one should take into account that the atomic structure–XPS spectrum relationship is often ambiguous and disputable, since the CEBEs of two atoms in different chemical environments can be the same. The inference of the (nano)­scaffold from the experimental XPS spectra is further obscured by the problem of overlapping XPS peaks in the spectrum. Golze et al.^4^ recognized the above points of concern associated with what they called “the backward route”–predicting to which molecular structure a given XPS spectrum belongs. A straightforward strategy aimed to remove this deficiency–consisting of theoretically predicting XPS spectra using combined machine learning techniques, DFT, and the GW approach (“the forward route”)–was proposed by them, yet only with regard to carbon-based nanomaterials.

Motivated by this interpretive challenge, I perform a comparative analysis of the CEBEs for a series of (TiO_2_)_*n*_ (*n* = 2–4) and Au_*m*_ (*m* = 4, 10, 20) clusters using the established ΔSCF framework to identify structural and electronic factors that influence their site-resolved core-level response. For each system (except Au_20_ due to its relatively extensive size), several geometrical isomers are considered to evaluate the dependence of CEBEs on the structural and electronic features.

The primary objective of this work is to shed light on how quantum size effects and the local bonding environment influence core-level binding energies in prototypical (sub)­nanoscale systems. To this end, I analyze how the calculated XPS signatures evolve with cluster size and with the nature of electronic screening. A further aim is to advance the modeling of CEBEs in heavy-element nanostructures, where scalar-relativistic effects are indispensable and spin–orbit coupling is, in principle, also relevant, while detailed site-resolved studies of core-level binding energies in finite nanomaterial systems remain scarce compared with the vast literature on molecules, surfaces, and bulk materials. Finally, I explore whether orbital-resolved eigenvalue shifts can be reduced to compact descriptors that correlate with CEBEs. Establishing such low-dimensional fingerprints would open another route toward data-driven models (e.g., machine–learning ones) for rapid prediction of site-specific core-level shifts and for screening larger nanostructure libraries.

It is important to note that the present work does not aim to introduce a universal computational protocol for simulating complete experimental XPS spectra; instead, it uses an established scalar-relativistic ΔSCF framework to analyze site-resolved CEBEs, and the associated orbital-resolved relaxation response and the spectra discussed below should therefore be understood as model envelopes used to connect the computed CEBE dispersion with its expected spectroscopic manifestation. The aggregated eigenvalue-shift descriptor is examined in the same spirit as an exploratory compact summary of the orbital-resolved relaxation response.

## 2 Computational Methods

The calculations were performed by means of the FHI-aims software^5^ (version 250320), an all-electron electronic structure package for computational materials science. Being based on numerically tabulated, atom-centered basis functions (numerical atomic orbitals, NAOs), FHI-aims provides significant precision in calculations^6^ and scalability,^7^ as NAOs require fewer basis functions compared with other types of basis sets; moreover, their strict localization allows for easy combination with linear scaling methods.^8^

In the case of Ti compounds, the basis sets were augmented with additional functions for the atoms on which a core–hole was imposed to facilitate the relaxation of all remaining electrons in the presence of the hole. For the Au compounds, such functions were omitted due to the lack of literature reports for such augmentations, to the best of the author’s knowledge.

All geometry optimizations and CEBE calculations were performed using the rSCAN functional.^9^ The SCAN functional,^10^ usually recommended for CEBE calculations,^11^ often led to numerical instabilities, especially in TiO_2_ clusters. However, rSCAN was also not entirely robust and, in a subset of cases, gave rise to severe SCF instabilities that could not be resolved, therefore rendering CEBE calculations for some subsets of geometries impossible.

For a limited set of representative Au_*n*_ and (TiO_2_)_*n*_ clusters, I also performed test calculations with the PBE functional. In these cases, PBE failed to qualitatively predict reported experimental trends connected to the size dependence,^12^ in line with earlier observations that semi-local GGAs can be unreliable for core-level spectroscopy,^3,13,14^ particularly when localization, screening, or self-interaction effects are significant.

Geometries were relaxed using the Broyden–Fletcher–Goldfarb–Shanno (BFGS) algorithm^15^ until the forces on all atoms were less than 5 × 10^–3^ eV·Å^–1^, and the accuracy of the converged electron density was chosen to be 1 × 10^–7^. The “tight” settings and third tier basis sets were employed for both geometry optimizations and total energy calculations to ensure convergence of core-level properties. Scalar-relativistic effects were included using the atomic ZORA (zero-order regular approximation) formalism, and nonself-consistent spin–orbit coupling correction (SOC) was initially contemplated as recommended for production settings but ultimately not included in the present production calculations, as it constitutes a merely postprocessing correction and does not modify the self-consistent electron density.^16^

All CEBEs reported here therefore correspond to scalar–relativistic ΔSCF *centroid* values of the experimental multiplet. These values, derived directly from SCF calculations, are meant to be degeneracy-weighted averages over the spin–orbit split 2*j* ± 1 components. This choice preserves methodological internal consistency without compromising the possible physical insights derived from density-based observables. Certainly, future work would undoubtedly benefit from incorporating SOC within a fully self-consistent relativistic framework.

I used value *l*_*hartree* = 8 and *radial*_*multiplier* = 8 keywords to enhance the accuracy of the multipole representation of the charge density and of the radial integration grid, respectively, together with explicitly specified dense angular grids up to the highest values possible for a given element employed in the present setup, including a tightened outer angular grid. The *deltascf*_*basis* keyword was used to calculate core-ionized densities for all the possible values of the index *m*, i.e., for the TiO_2_ clusters *m* ∈ {−1, 0, 1}, and for the Au compounds *m* ∈ {−3, ..., 3}. It is important to note that here *m* is used as a label distinguishing the selected real-orbital component within a given *l*-subshell so as to resolve the different angular components of the core–hole state, rather than as a strict magnetic quantum number in the atomic-physics sense. The default energy convergence settings were applied, and the constrained core-ionized Kohn–Sham states were chosen as the ones with the largest projection coefficient onto the selected basis set function.

The statistical analysis was performed by means of in-house Python scripts using such libraries as pandas,^17^ NumPy,^18^ SciPy,^19^ Matplotlib,^20^ and pymatgen.^21^

## 3 Systems Studied

TiO_2_- and Au-based nanostructures represent two complementary systems for investigating quantum effects: the former as a prototypical transition-metal oxide with mixed ionic–covalent bonding character and the latter as a paradigmatic metal cluster where relativistic and electron delocalization effects dominate.

Nanostructured systems, particularly metal and oxide clusters, exhibit markedly different properties from their extended bulk analogues.^22^ Reduced coordination numbers, surface-dominated environments, and discrete energy-level structures contribute to their unique electronic behavior. Consequently, the interpretation of XPS spectra in nanomaterials requires a more nuanced approach, often necessitating accurate first-principles calculations to disentangle initial state effects (electronic structure prior to photoemission) from final state effects (relaxation and screening after ionization).

### 3.1 TiO_2_ Nanoclusters

The TiO_2_ clusters were chosen as prototypical molecular fragments of bulk rutile- and anatase-type compounds, capturing the key Ti–O linkages in a computationally relatively undemanding, finite form. Within this size range, the compounds already display a variety of local environments, including differently coordinated Ti centers and both terminal and bridging oxygen atoms, which makes them suitable for sampling chemically distinct core sites within a single, well-defined structural motif. They can thus be regarded as minimal models for probing how local Ti–O coordination and cluster size influence core-level binding energies and XPS signatures in transition-metal oxides while keeping the cost of systematic ΔSCF calculations manageable. Figures 1 and 2 depict selected optimized geometries of TiO_2_ clusters.

**Figure 1:**
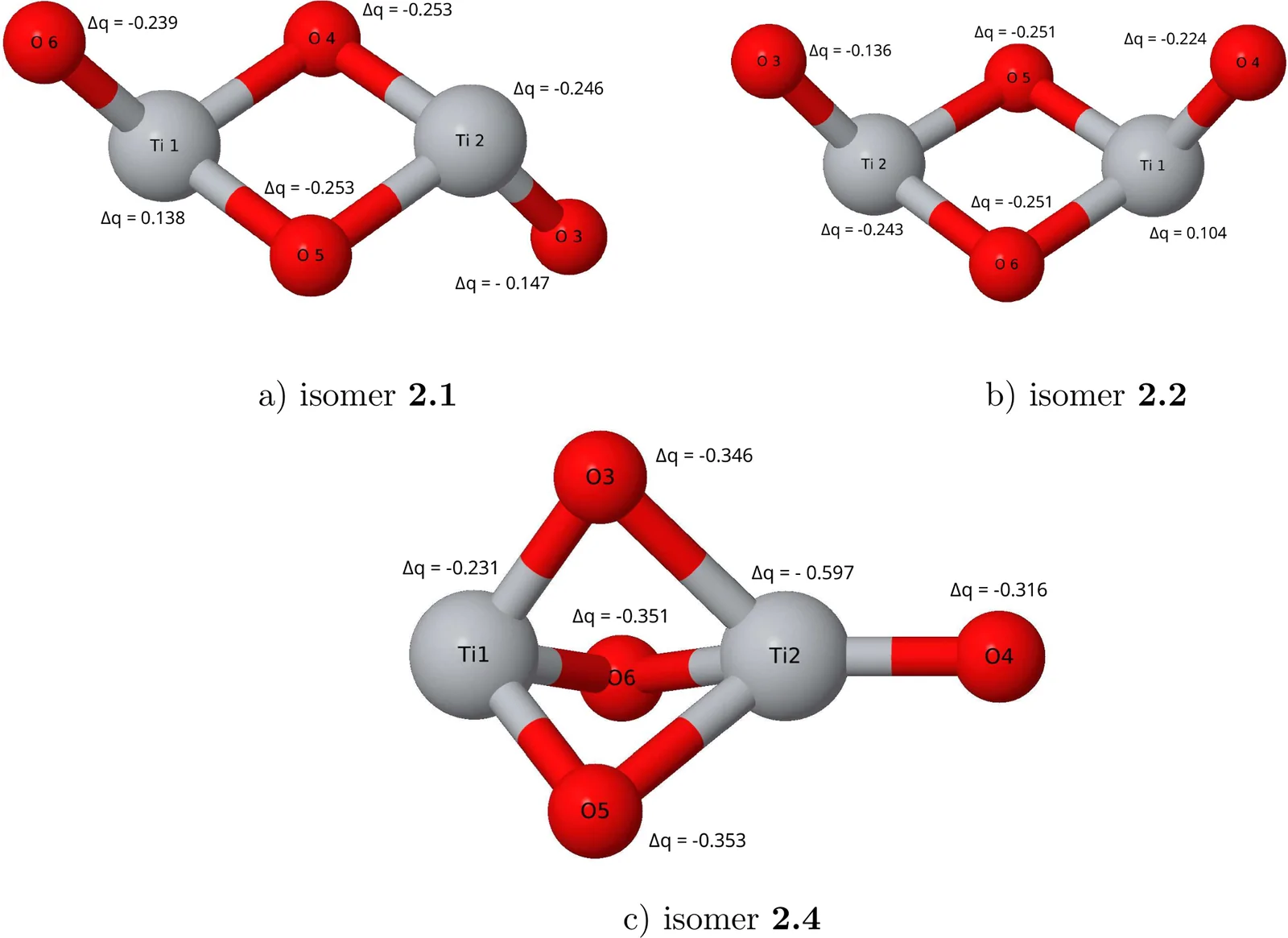
Optimized geometries of selected TiO_2_ dimer isomers used in the discussion below. Atom labels are shown explicitly. The Δ*q* values are included to avoid duplicating a separate structural figure in Section 4.2.3 where they are discussed. The charge values correspond to *m* = 0 at the Ti1 atom in **2.1** and **2.2** and at the Ti2 atom in **2.4**.

**Figure 2:**
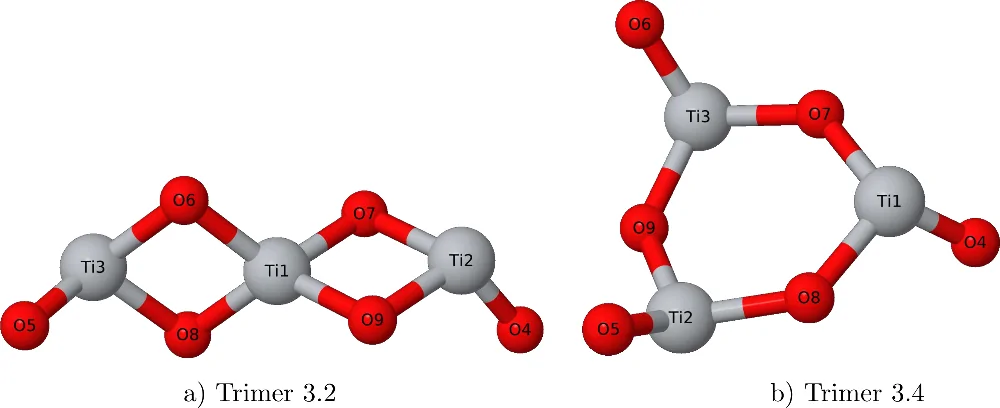
Optimized geometries of the (TiO_2_)_3_ clusters **3.2** and **3.4**. Ti atoms are labeled explicitly; O atoms are numbered for reference in the subsequent charge-analysis discussion.

### 3.2 Au Nanoclusters

I selected the Au particles as representative gold clusters, spanning the transition from small, strongly quantum-confined systems to larger species with incipient bulk-like character. Au_4_ serves as the arguably smallest nontrivial cluster with structural isomers with diverse spatial arrangements; Au_10_ is an intermediate-size structure combining low- and higher-coordinated sites, and Au_20_ is a widely studied elsewhere (see, e.g., Tsukuda et al.^23^) “magic-number” cluster whose geometry and electronic structure are even closer to those of bulk-like gold nanoparticles while still remaining computationally tractable within the cumulative cost of the present all-electron, site-resolved ΔSCF protocol, allowing an assessment of how core-level binding energies and relaxation patterns evolve as the system approaches the metallic regime. Figure 3 depicts selected optimized geometries for Au_4_ and Au_10_ isomers.

**Figure 3:**
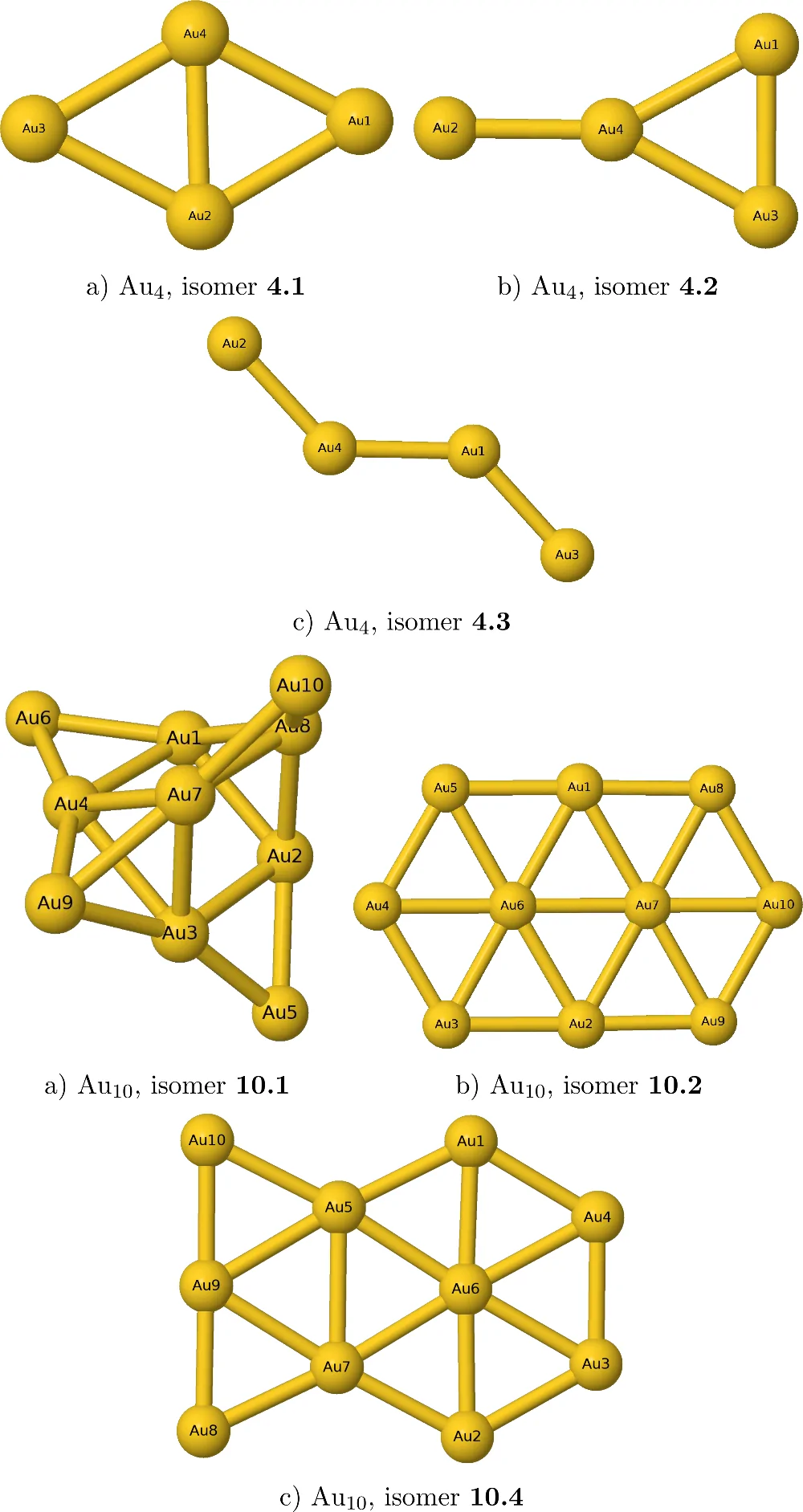
Selected geometries of the Au_4_ and Au_10_ isomers considered in this work. Atom numbers are shown.

### 3.3 Structural Models of the Investigated Clusters

The geometries under study were mostly published previously: the coordinates used as the input geometries for TiO_2_ oligomers were published by Olvera-Neria et al.;^24^ the initial coordinates for Au_4_ are available in Kim et al.;^25^ for Au_10_ nanoclusters, see Nguyen et al.;^26^ and the geometry for Au_20_ was created by hand based on the work of Lai et al.^27^ The numbers of the compounds in this work try to follow the numbering given by the respective authors (e.g., 2.**4** corresponds to EA**4** by Olvera-Neria). All distinct geometries of the lowest-energy isomers are available in the .xyz format in the Supporting Information (Section S1).

## 4 Results and Discussion

This section is organized by increasing level of electronic-structure detail. I first discuss the distributions of ΔSCF CEBEs and their size dependence because these quantities define the site-resolved energetic information from which the model XPS envelopes are constructed. I then use the broadened spectra to summarize how the calculated CEBE distributions translate into qualitative line-shape regimes. Finally, I analyze the orbital-resolved Kohn–Sham eigenvalue shifts underlying the core–hole relaxation response and examine whether this high-dimensional information can be compressed into an exploratory scalar descriptor.

### 4.1 CEBEs and Cluster-Size Dependence

Prior to CEBE calculations for the investigated Ti and Au compounds, the number of symmetrically equivalent element atoms in a given cluster was assessed via an in-house python script, and the first atom of each (in)­equivalence category was chosen as the representative one. For each of these representatives, CEBEs were computed using the standard ΔSCF approach used repeatedly elsewhere:^1,11,28^ two energy calculations were carried out for the optimized geometries–one for the neutral ground state (GS) and one for the spin-polarized, core-ionized final state (core–hole or CH) in which the occupation number of a selected core orbital of the given atom *A* was reduced by one electron from the spin channel 1 (arbitrarily selected) according to the equation:

1 $$CEBE\left(A\right)=E{\left(A\right)}^{\left(\mathrm{core}\text{−}\mathrm{hole}\right)}-E{\left(A\right)}^{\left(\mathrm{GS}\right)}$$

In both families of systems, most of the CEBEs for Au and Ti core subshells (4f ≈ 90 eV and 2p ≈ 465 eV, respectively) fall within the ranges that slightly deviate from the bulk values but still appear reasonable given their molecular and nanocluster environments. Yet, the trends with size and local environment are markedly different.

Figures 4 and 5 depict the distributions of the computed CEBEs after statistical processing. Each box plot reflects the spread of CEBEs over all symmetry inequivalent atoms in a given cluster. Together, these data allow an assessment of both the intracluster heterogeneity of CEBEs and their systematic evolution with cluster size.

**Figure 4:**
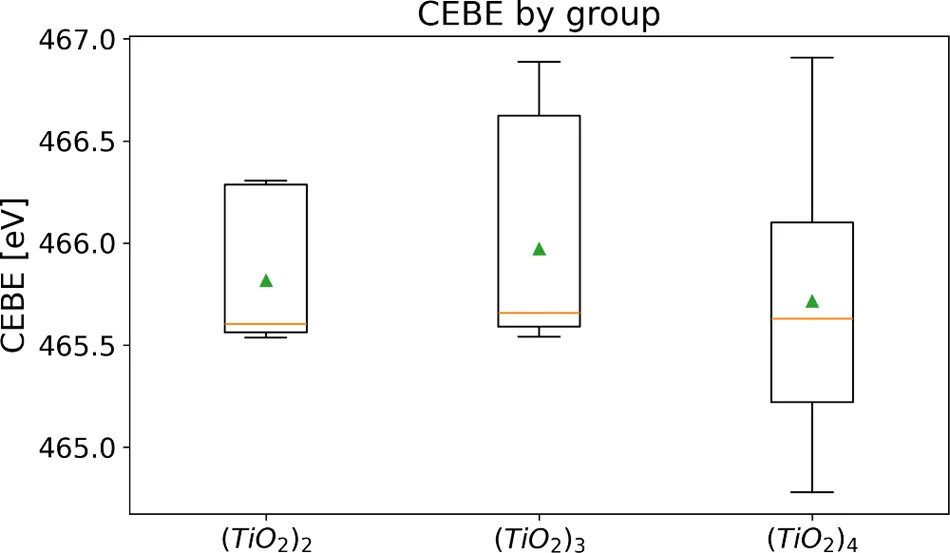
Boxplot of CEBE distributions for TiO_2_ oligomers after excluding the outliers. Green triangles indicate the mean and the orange lines, the median.

**Figure 5:**
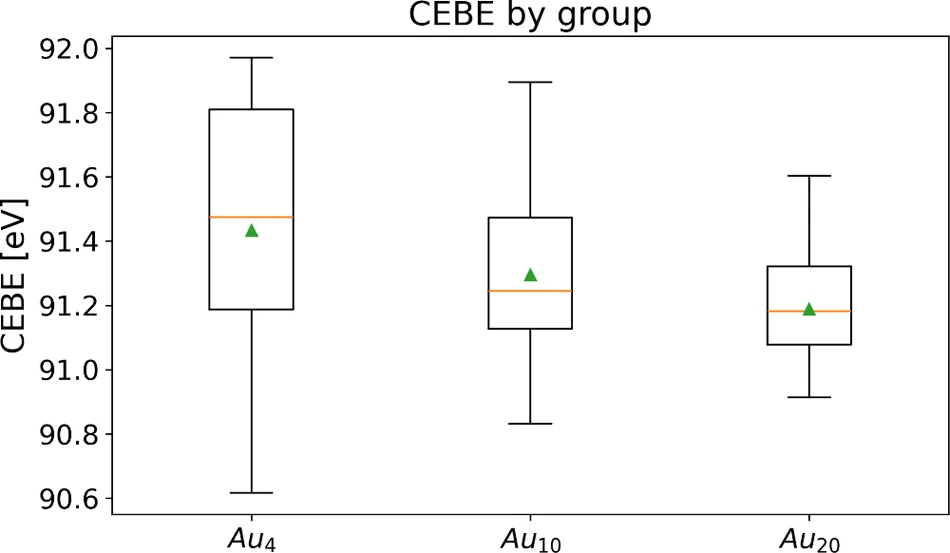
Boxplot of CEBE distributions for Au clusters after excluding the outliers. Green triangles indicate the mean and the orange lines, the median.

For each cluster, I characterize the distribution of CEBEs by its median value after excluding the outliers, which serves as an estimate of a “typical” core level energy within the cluster. As the cluster size increases, the relative contribution of surface atoms with more bulk-like local coordination environments grows, whereas the relative weight of strongly undercoordinated edge- and corner-like environments decreases. At the same time, photoelectrons originating from deeper layers are expected to have a lower probability of escaping without inelastic scattering and therefore should contribute only weakly to the measured spectrum. Under these assumptions, the median CEBE is expected to converge toward the bulk value in the large-cluster limit. All of the calculated CEBE values are available in the Supporting Information (Tables S1–S8).

#### 4.1.1 TiO_2_ Oligomers: Size-Independent CEBEs

For the TiO_2_ series, Figure 4 depicts the box plots summarizing the deterministic distributions of site-resolved CEBEs obtained for the symmetry-inequivalent Ti atoms in each cluster. The three distributions overlap strongly, and their median values are 465.60, 465.66, and 465.62 eV for (TiO_2_)_2_, (TiO_2_)_3_, and (TiO_2_)_4_, respectively, which indicates their confinement to a range of only ≈0.05 eV. The corresponding means differ somewhat more (465.81, 465.97, and 465.69 eV) but still remain within ≈0.28 eV.

This qualitative impression is supported by a Theil–Sen analysis of the median CEBEs as a function of cluster size *n*. The estimated slope is 0.008 eV per TiO_2_ unit with a 95% confidence interval ranging from −0.04 to 0.05 eV. Within this range, the calculated CEBEs do not provide evidence for a systematic size dependence between *n* = 2 and *n* = 4. This absence of a clear trend is consistent with experimental reports for larger anatase nanoclusters, where the binding energy likewise remains essentially unchanged, suggesting that particle-size effects do not contribute substantially.^29^

At the same time, the intracage spread is non-negligible and increases across the series. The standard deviation grows from about 0.36 eV for (TiO_2_)_2_ to about 0.62 eV for (TiO_2_)_4_, reflecting the growing diversity of local Ti environments and the associated variation in the site-resolved CEBEs. Thus, the TiO_2_ clusters exhibit substantial local heterogeneity without a corresponding monotonic shift in the cluster-level central tendency. The practical spectroscopic consequence of this distinction is addressed below through Gaussian-broadened model XPS line shapes.

#### 4.1.2 Au Clusters: A Systematic, Size-Dependent CEBE Downshift

In contrast to the TiO_2_ series, the Au clusters show a clear downward shift in the CEBE distributions with increasing size (Figure 5). The median values decrease from 91.47 eV for Au_4_ to 91.25 eV for Au_10_ and 91.18 eV for Au_20_, corresponding to an overall shift of ∼0.29 eV across the present size range. The mean values follow the same trend (91.43, 91.30, and 91.19 eV), and the overlap between the distributions is visibly reduced for larger clusters.

A Theil–Sen regression of the median CEBE as a function of cluster size yields a slope of −0.018 eV per atom with a 95% confidence interval from −0.038 to −0.006 eV. Since this interval does not include zero, the data support a monotonic size-dependent downshift over the present range. The intracluster dispersion remains appreciable with standard deviations of about 0.37, 0.26, and 0.20 eV for Au_4_, Au_10_, and Au_20_, respectively, but it decreases as the cluster grows.

These results are qualitatively consistent with experimental observations for small Au clusters, which likewise show decreasing binding energies with increasing size together with line width broadening exceeding the bulk value.^12^ In this sense, the calculated site-resolved CEBE distributions may be viewed as a microscopic counterpart of the inhomogeneous broadening seen experimentally, while the overall downward shift of the median reflects the progressive buildup of more efficient metallic screening and electronic delocalization. The practical manifestation of these calculated distributions in terms of broadened spectral envelopes is illustrated in the following subsection through model XPS line shapes. The strong intracluster dispersion and its sensitivity to local coordination, strain, and morphology are in line with the core level shifts driven by electrostatic potential change identified by Tal et al.^30^

#### 4.1.3 From Calculated CEBEs to Model XPS Line Shapes

To connect the calculated CEBEs more directly with experimentally observable quantities, I constructed model XPS line shapes by Gaussian broadening of the discrete set of calculated CEBEs; full width at half-maximum (fwhm) was chosen to be 0.4 eV. All computed *m*-labeled components were retained explicitly rather than averaged over for a given atomic site so as to preserve the full structure of the raw ΔSCF results and avoid introducing an additional reduction step at the spectral-construction stage. The resulting curves should therefore be understood as model spectral envelopes derived directly from the complete calculated CEBE distributions, rather than as claims that the individual *m*-resolved contributions would necessarily be separately resolvable in the experiment.

Inspection of the complete set of Gaussian-broadened model spectra shows that the calculated CEBE distributions lead to several types of spectral envelopes. Compact CEBE distributions, as observed most clearly for Au_20_ and for several Au_10_ isomers, collapse after broadening into nearly single-feature envelopes. Broader or more asymmetric CEBE distributions, especially in Au_4_ and selected TiO_2_ oligomers, generate visibly asymmetric line shapes or shoulder-like features. Finally, strongly heterogeneous Ti environments may produce more clearly separated subbands, most notably when atypical Ti sites are present. Thus, the broadened spectra confirm that the site-to-site CEBE dispersion is not only a statistical property of the calculated data set; depending on its magnitude relative to the assumed line width, it may appear as unresolved inhomogeneous broadening, as an asymmetric envelope, or as a visible internal structure in the model XPS line shape. All created figures for all isomers are available at 10.5281/zenodo.20067444.

The qualitative regimes identified from the complete spectral data set are summarized in Tables 1 and 2, where representative envelopes are shown for each recurring line shape type. However, the representative spectra are not intended to assign every broadened component to a separately resolvable experimental peak. Rather, they summarize how the calculated site-resolved CEBE distributions translate into different qualitative model-envelope regimes, ranging from unresolved inhomogeneous broadening to clearly separated subbands.

**Table 1:**
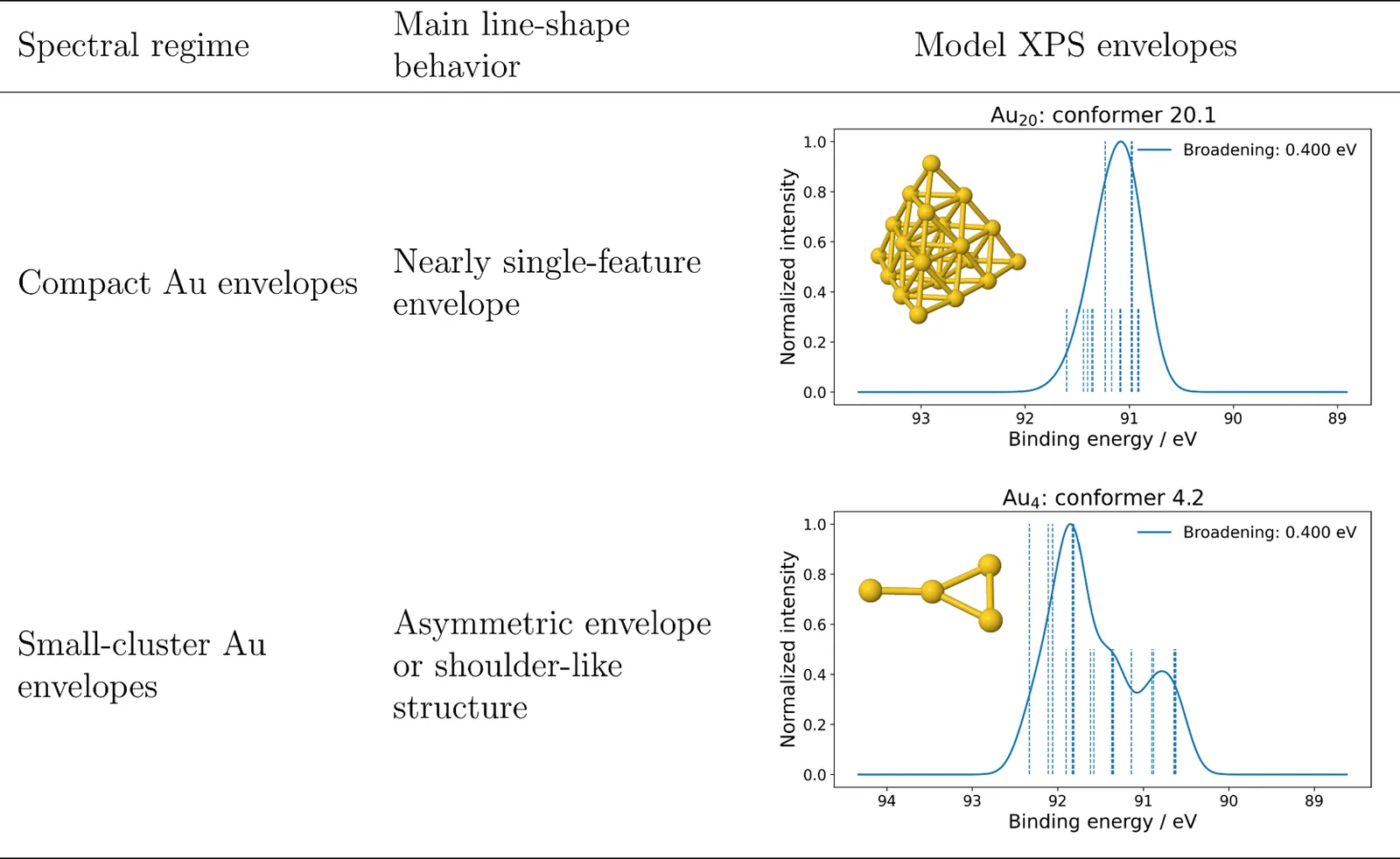
Qualitative Classification of the Model XPS Envelopes Obtained for Au Clusters^a^

a FWHM = 0.4 eV.

**Table 2:**
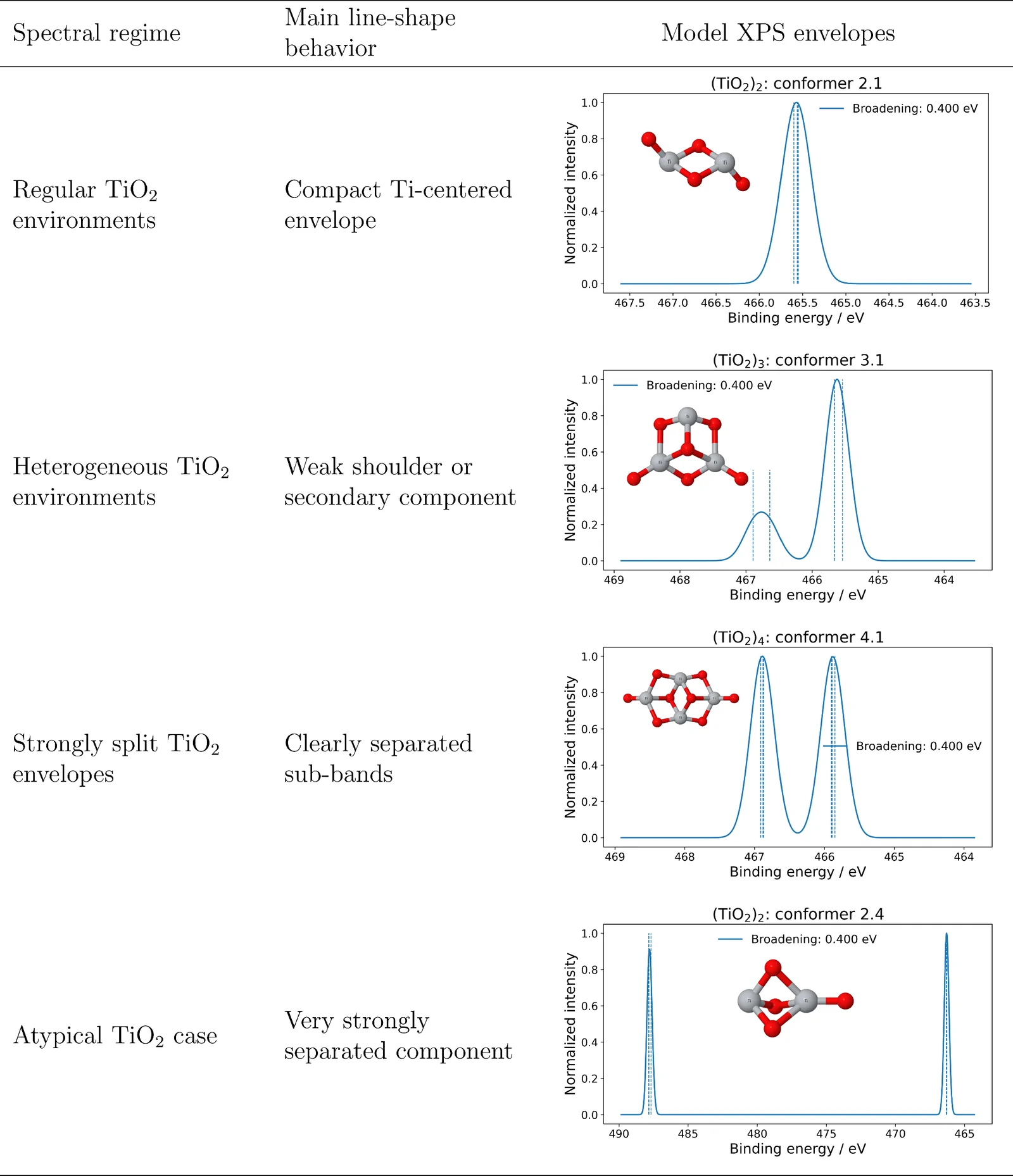
Qualitative Classification of the Model XPS Envelopes Obtained for TiO_2_ Clusters^a^

a FWHM = 0.4 eV.

Having thus connected the site-resolved CEBE distributions to model XPS envelopes, I now turn to the orbital-resolved response that underlies these total energy shifts.

### 4.2 Orbital-Resolved Eigenvalue Shifts upon Core Ionization

#### 4.2.1 Eigenvalue Shifts as Fingerprints of the Local Chemical Environment

While ΔSCF total-energy differences provide access to absolute, site-resolved CEBEs, the underlying Kohn–Sham (KS) eigenvalue shifts contain complementary information on how the electronic structure reorganizes in response to a localized core–hole. To quantify this response, I analyzed for each ionized cluster *k* the changes in all occupied eigenvalues associated with it

2 $$\Delta \varepsilon_{i}^{k}=\varepsilon_{i}^{k\left(CH\right)}-\varepsilon_{i}^{k\left(GS\right)}$$

where $\varepsilon_{i}^{k\left(GS\right)}$ and $\varepsilon_{i}^{k\left(CH\right)}$ denote the *i*-th ground-state and core–hole KS eigenvalues, respectively.

Creating a core–hole introduces a perturbation in the effective Kohn–Sham potential:

3 $$\delta V_{e\mathrm{ff}}\left(r\right)=V_{e\mathrm{ff}}^{CH}\left(r\right)-V_{e\mathrm{ff}}^{GS}\left(r\right)$$

which is predominantly localized because it is centered on the ionized atom. In a simplified, first-order perturbative picture, the corresponding eigenvalue shift of a ground-state Kohn–Sham orbital ϕ_*i*_ can be interpreted approximately as

4 $$\Delta \varepsilon_{i}\approx \left\langle \phi_{i}\left|\delta V_{e\mathrm{ff}}\right|\phi_{i}\right\rangle =\int \delta V_{e\mathrm{ff}}\left(r\right)|\phi_{i}\left(r\right){|}^{2}d^{3}r$$

showing that Δε_*i*_ is, in this picture, controlled by how much electronic density associated with orbital *i* is located in the vicinity of the core–hole. Orbitals localized on the ionized atom (deep core, semi–core, and strongly bonded valence states) are therefore expected to experience substantial shifts, whereas more remote or weakly coupled states should be affected only weakly. Since δ*V*_eff_(**r**) is localized mainly around the ionized site, its shape is determined primarily by the initial-state contributions of the local chemical environment–the immediate coordination, bond lengths, oxidation state, and ligand types–and is largely insensitive to the more distant parts of the cluster. Additionally, final-state effects such as orbital relaxation upon core–hole creation go beyond the approximate expression in eq 4 and further influence the exact value of Δε_*i*_.

For isolated finite clusters, the vacuum level provides a well-defined reference for absolute orbital energies. In the present analysis, however, I do not use the Kohn–Sham eigenvalues as absolute spectroscopic observables; they are used to compare the relative orbital-resolved response induced by a localized core–hole. I therefore introduce an internal alignment convention in which all eigenvalues are referenced to the deepest 1s level of the ground-state atom in each calculation. This choice is motivated by the fact that the 1s orbital is the deepest core state and is therefore expected to undergo the smallest relative change between the ground-state and core–hole solutions. This removes global offsets from the comparison of Δε_*i*_ patterns and emphasizes relative, subshell-resolved changes in the electronic structure; this internal alignment affects only the analysis of the Kohn–Sham eigenvalue shift patterns and does not affect the ΔSCF CEBEs.

Since the atom-resolved CEBE tables quantify the final scalar binding-energy shifts but they do not show how the electronic structure reorganizes after core ionization, the orbital-resolved Δε_*i*_ plots are therefore used here as a complementary diagnostic of the screening response. In particular, they reveal whether the core–hole perturbation produces an almost rigid shift of inner shells or a more selective reorganization of outer-core and valence states.

Indeed, the present data suggest that sites sharing a similar local environment give rise to very similar patterns of Δε_*i*_ once all shifts are measured relative to the same ground-state reference. In particular, Ti atoms with the same number and type of O neighbors and comparable Ti–O distances exhibit Δε_*i*_ ladders that remain very similar in both overall structure and magnitude with the dominant shifts typically differing by no more than about 0.1 eV, even when they belong to different (TiO_2_)_*n*_ clusters, and likewise for Au atoms in different Au_*n*_ clusters. In this sense, the Δε_*i*_ spectrum–especially that of the core and semi-core subshells of the ionized atom–acts as a compact fingerprint of the local chemical environment and screening behavior at a given site: when two atoms experience closely similar local coordination shells, the core–hole perturbation and the resulting redistribution of electronic density are nearly the same and so are the corresponding eigenvalue-shift patterns. In contrast, the response of the valence subshells of all atoms typically shows much more variation between sites, further corroborating their role as sensitive probes of long-range screening patterns. All of the orbital-resolved Δε plots and heatmaps are available at 10.5281/zenodo.20067444.

In the following comparative figures, the scatter plots show the full aligned Δε_*i*_ profiles, whereas the heatmaps are used as an auxiliary measure to emphasize small orbital-by-orbital differences relative to the explicitly stated reference calculation.

#### 4.2.2 TiO_2_ Clusters

For the TiO_2_ clusters, the Δε_*i*_ patterns are remarkably regular across the entire series. After alignment to the deepest 1s­(GS) level, the deep core and semicore–shells form an almost rigid ladder, whereas most of the variation is confined to the valence region. This indicates that the core–hole acts predominantly as a local perturbation whose detailed response is governed mainly by the immediate Ti–O coordination environment.

Figure 6 depicts the corresponding eigenvalue-shift plots for the **2.4** isomer (cf. Figure 1). For Ti sites sharing closely similar local coordination environments across this dimer series, the resulting Δε_*i*_ ladders remain very similar in both overall structure and magnitude with very small differences in values, supporting the notion of a compact fingerprint of the local perturbation.

**Figure 6:**
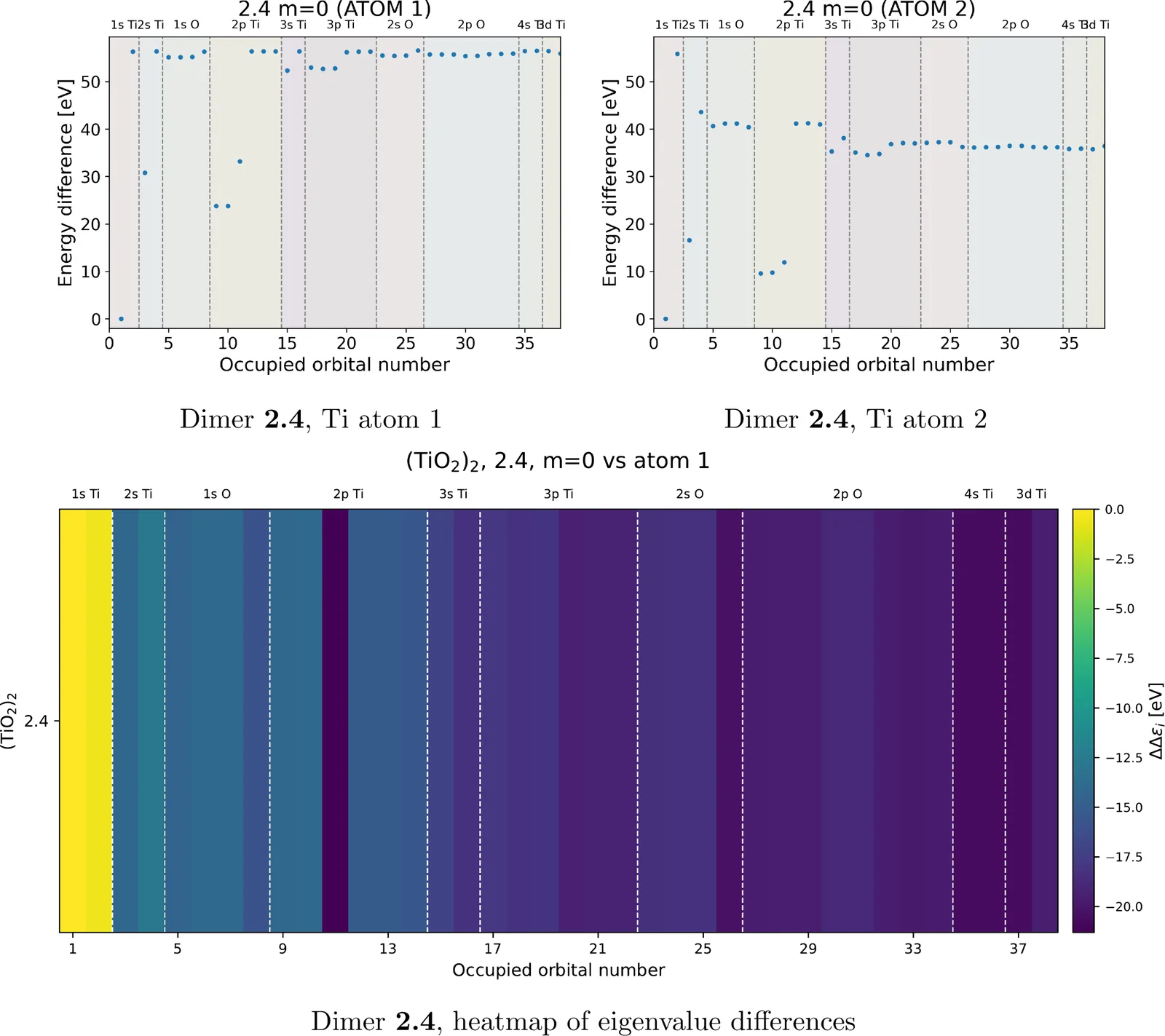
Δε_*i*_ as a function of orbital index for the two inequivalent Ti atoms in **2.4** for *m* = 0 (i.e., isomer **2.4**, atom A1, *m* = 0 and **2.4**, A2, *m* = 0). Different colors indicate different atomic subshells. The heatmap shows the orbital-by-orbital differences between the two calculations on the same scale using (**2.4**, A1, *m* = 0) as the reference.

One Ti site deviates qualitatively from this generic pattern. As shown in Figure 6, the Ti2 atom in **2.4** exhibits a markedly compressed Δε_*i*_ ladder and an anomalously large CEBE of about 487 eV compared with 465–466 eV for the other Ti sites (cf. Table S1 in the Supporting Information). A localization check based on per-atom spin populations confirms that the missing electron is associated with the intended Ti2 site and is dominated by the *l* = 1 contribution for all values of *m*, consistent with a Ti 2p core–hole. This anomalous site is discussed separately below.

#### 4.2.3 Anomalous Ti2 Site in (TiO_2_)_2_

For all other Ti sites in the dimers considered, the eigenvalue-shift pattern forms a well-defined ladder followed by a more dispersed set of valence levels. In contrast, for Ti2 in **2.4**, the entire ladder is compressed and shifted toward smaller absolute values. This indicates substantially stronger screening of the core–hole δ*V*_eff_(**r**) perturbation at this site. The effect is essentially independent of the choice of the value of the index *m*, which suggests that it reflects a genuine change in the screening mechanism rather than a simple orientation effect.

The Mulliken Δ*q* = *q* ^CH^ – *q* ^GS^ values support this interpretation (cf. Table S9 in the Supporting Information). For the nonanomalous Ti sites, core–hole formation increases the local electronic population on the ionized Ti by ≈0.1–0.35 e̅ . For Ti2 in **2.4**, however, the corresponding increase is ≈0.6 e̅ with charge drawn both from the neighboring Ti and from the surrounding O atoms. This points to a much more localized and efficient screening response, consistent with a Ti^3+^-like final state and with the strongly compressed Δε_*i*_ ladder.

The origin of this behavior can be traced to the local geometry of **2.4** (Figure 1). In **2.1** and **2.2**, each Ti atom is coordinated by one shorter Ti–O bond (∼1.66 Å) and two longer ones (∼1.84 Å), giving a reasonably balanced local environment. In **2.4**, in contrast, Ti2 retains one short Ti2–O4 bond (1.67 Å) but the remaining Ti2–O distances are elongated to about 1.99 Å. This strongly uneven coordination weakens delocalization along several Ti–O directions and favors a more localized screening response once the core–hole is created. The result is both the unusually large CEBE and the compressed eigenvalue-shift profile of Ti2. Within the statistical analysis of Section 4.1, Ti2 in **2.4** is therefore treated as an electronically atypical site and excluded from the trend analysis.

#### 4.2.4 Topology Effects in TiO_2_ Trimers

The TiO_2_ trimers show that the Δε_*i*_ patterns depend not only on the immediate Ti–O coordination but also on how the ionized site is embedded in the extended Ti–O–Ti network. As illustrated in Figure 2, Ti2 in **3.2** and Ti1 in **3.4** appear chemically similar at the level of their first coordination shell: both are surrounded by three O ligands, two bridging and one terminal. In contrast, Ti1 in **3.2** plays the role of a more central connector between two TiO_3_ fragments. A very similar Δε_*i*_ pattern would therefore be expected for Ti2­(**3.2**) and Ti1­(**3.4**) if the eigenvalue shifts were sensitive to the first coordination shell only.

The corresponding differences in the eigenvalue shifts are summarized in the heatmap shown in Figure 7, where Ti1­(**3.2**) is used as the reference. Ti1­(**3.4**) appears overall closer to Ti1­(**3.2**) than Ti2­(**3.2**), whereas Ti2­(**3.2**) displays larger and more structured deviations across several orbital regions, including the valence part of the spectrum. Thus, the map supports the view that similarity of the first coordination shell alone is not sufficient to guarantee a similarly shaped Δε_*i*_ pattern. In this case, a wider color scale was adopted to make the magnitude of the differences more clearly visible.

**Figure 7:**
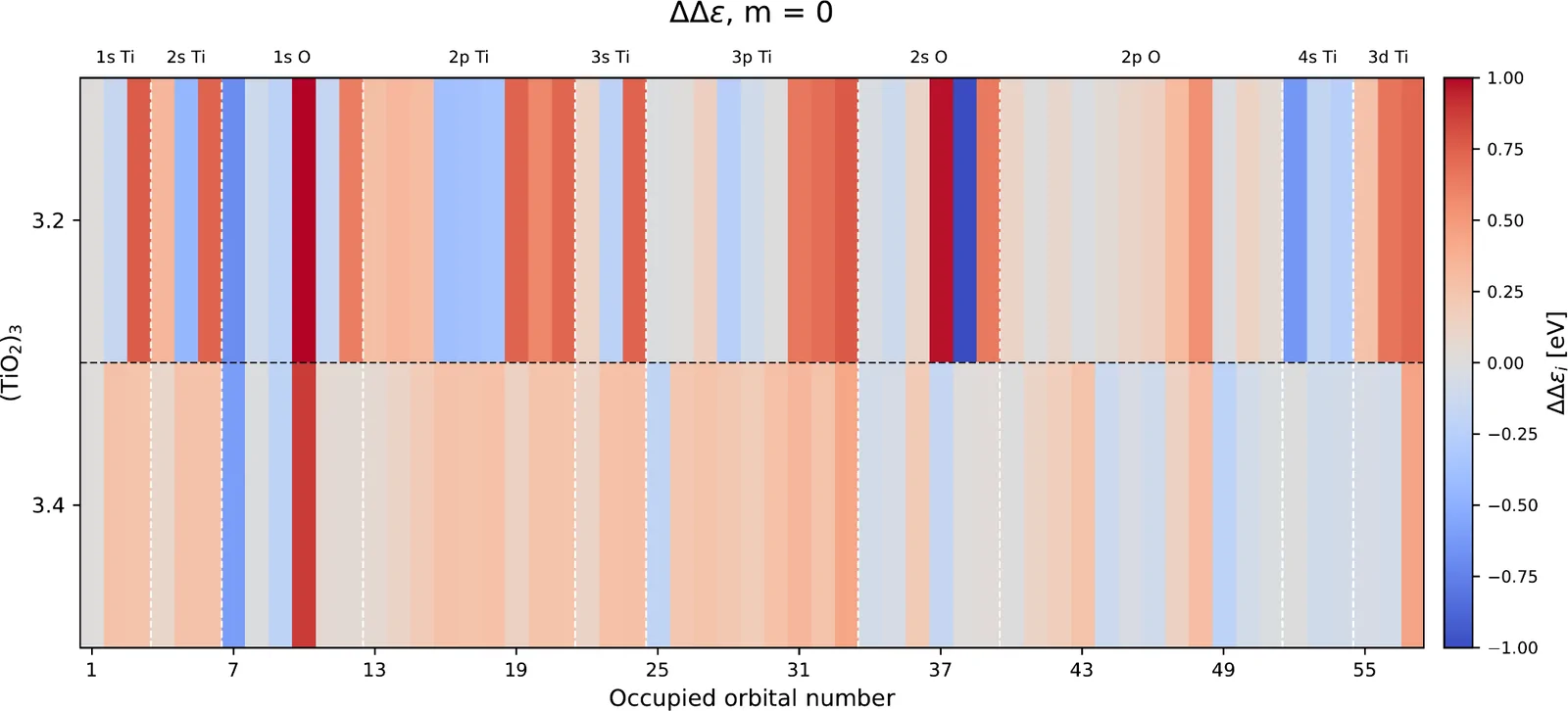
Δε_*i*_ heatmap for the *m* = 0 Ti sites: (**3.2**, A2) and (**3.4**, A1), shown as differences relative to (**3.2**, A1). Rows correspond to individual core–hole calculations.

This picture is further supported by the Mulliken Δ*q* values in Table S10 (see Supporting Information). For a core–hole on Ti1 in **3.2** or on Ti1 in **3.4**, the additional screening charge is redistributed in a relatively balanced way over the neighboring Ti and O atoms. For Ti2 in **3.2**, however, the screening becomes noticeably more asymmetric with one side of the chain contributing much more strongly than the other. This is consistent with the structural role of Ti2­(**3.2**) as an edge site, whereas Ti1­(**3.2**) and Ti1­(**3.4**) act as internal connectors.

Overall, these observations suggest that the valence part of the Δε_*i*_ spectrum is sensitive not only to the first coordination shell but also to the topology of the Ti–O–Ti scaffold and to the number and symmetry of the available screening pathways. In contrast, the core and semicore ladders remain noticeably more rigid, consistent with the more local character of the corresponding orbitals.

#### 4.2.5 Atom-Dependent Core-Level Shifts in the TiO_2_ Clusters

To make the core-level shifts explicit, each symmetry-inequivalent site was characterized by the median of its *m*-resolved CEBEs (denoted by $\overset{\sim }{CEBE}$ ) and by the corresponding site shift $\Delta \overset{\sim }{CEBE}$ defined relative to the lowest median CEBE within the same isomer (cf. Table S11 in the Supporting Information). These descriptors were derived from the optimized geometries using distance cutoffs obtained from the interatomic-distance distributions of the entire data set: for each relevant pair type, all possible corresponding interatomic distances were collected and the cutoff was then defined as the first minimum separating the first coordination shell from the subsequent part of the distance distribution. This procedure yielded Au–Au, Ti–O, and Ti–Ti cutoffs of 3.275, 2.275, and 3.025 Å, respectively, avoiding a manual tuning of the thresholds for individual systems. The descriptors should be thus understood as practical measures of the local coordination shell rather than as unique bond definitions.

For the TiO_2_ clusters, the atom-dependent CEBE shifts confirm that the site energetics cannot be reduced to the nominal Ti coordination number alone. The automatically extracted descriptors show that formally similar O_3_ environments may still differ substantially in CEBE depending on the balance between terminal and bridging O ligands and on the wider embedding of the Ti site in the Ti–O–Ti scaffold. In **3.2**, for example, Ti2, coordinated by one terminal and two bridging O atoms (denoted as O_2_), has the lowest median CEBE, whereas the more fully connected O_4_ site Ti1 lies 0.133 eV higher. A similar tendency appears in **4.10**, where Ti4, again coordinated by one terminal and the bridging O_2_ ligands, defines the minimum, while the formally also O_3_-coordinated Ti2 is shifted upward by 1.189 eV. At the same time, **4.12** shows that even the simple O_3_/O_4_ distinction is insufficient on its own: here, the O_4_ site Ti3 lies 1.563 eV below the fully bridged O_3_ site Ti2. Thus, the decisive factor is not only the number of O neighbors but also the detailed balance of terminal versus bridging ligands together with the way in which the site is embedded in the larger Ti–O–Ti network. The exceptional Ti2 site in **2.4**, whose median CEBE exceeds that of Ti1 by more than 21 eV, remains a clear outlier and should be regarded as a distinct coordination class rather than as a smooth continuation of the remaining Ti environments.

#### 4.2.6 Au Clusters

For Au_4_, Au_10_, and Au_20_ clusters, the orbital-resolved eigenvalue shifts induced by a 4f core–hole follow the same broad pattern as in the TiO_2_ clusters. Figure 3 shows the representative Au_4_ and Au_10_ isomers considered in the discussion below, and Figure 8 shows the selected eigenvalue-shift patterns corresponding to the isomer **4.1** of the Au_4_ cluster. After alignment to the deepest 1s­(GS) level, the deep core responds almost rigidly within a given subshell, the valence states show the largest reorganization, and the partially depopulated 4f shell occupies an intermediate position.

**Figure 8:**
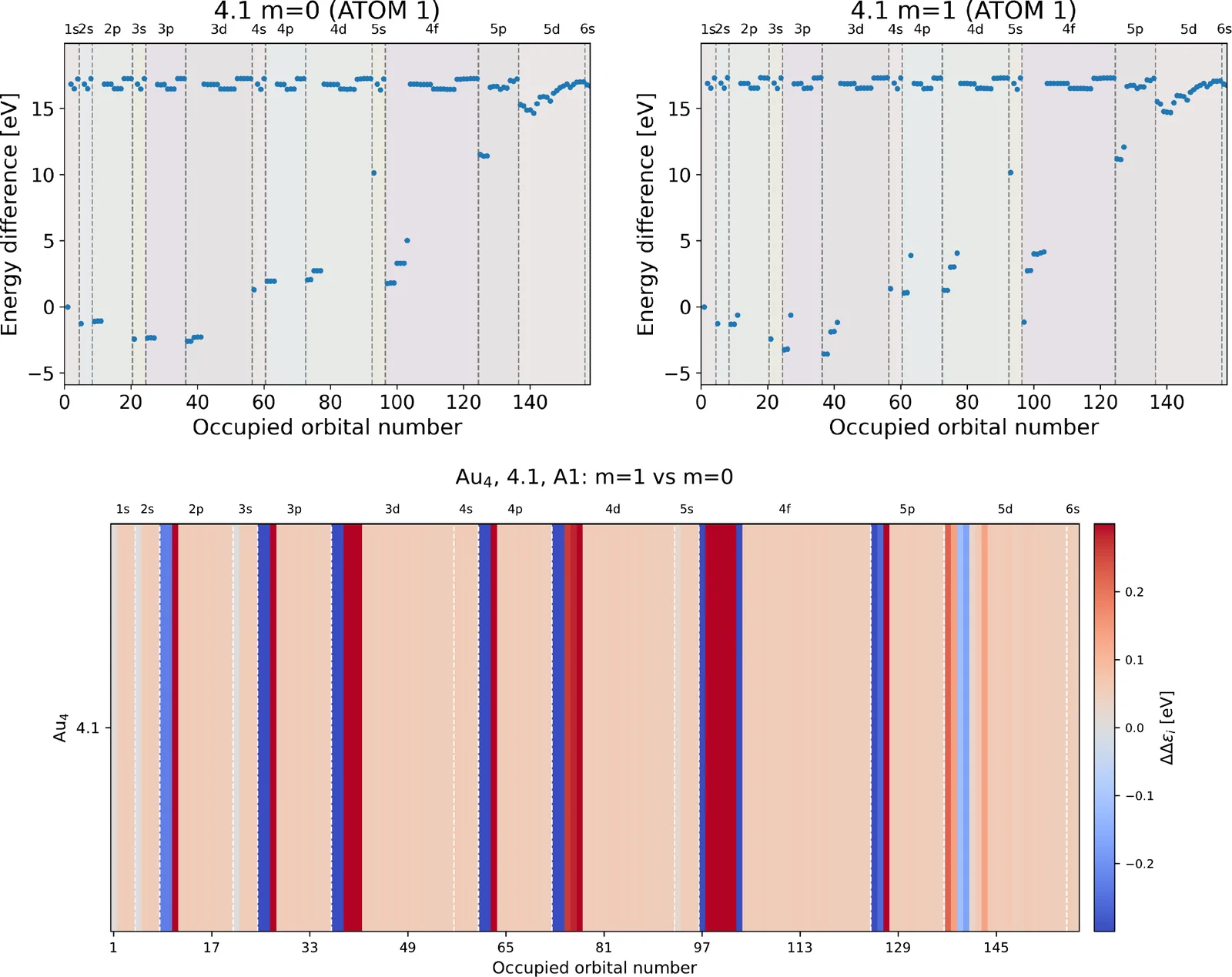
Δε_*i*_ as a function of orbital index for Au_4_ (**4.1**, A1, *m* = 0 and *m* = 1). The heatmap shows the orbital-by-orbital difference between (**4.1**, A1, *m* = 1) and (**4.1**, A1, *m* = 0) taken as the reference.

In contrast to TiO_2_, however, the Au clusters display a more clearly resolved dependence on the chosen *m*-index of the core–hole. This is consistent with the greater angular complexity and spatial extent of the 4f shell being able to sample regions where the anisotropic part of the local potential remains non-negligible. Accordingly, the different *m*-instantiations of the core–hole generate slightly different but systematic Δε_*i*_ patterns.

At the same time, the core and 4f parts of the Au spectra remain highly reproducible across chemically different sites and clusters (cf. Figure 9). When grouped by subshell, they form a small set of recurring motifs with point-to-point differences typically of the order of only 0.01–0.1 eV, i.e., very small compared with the overall shifts of order 20 eV. This again indicates that the dominant contribution to the deep-shell response is local, while the details of the bonding environment enter mainly as a weaker anisotropic perturbation.

**Figure 9:**
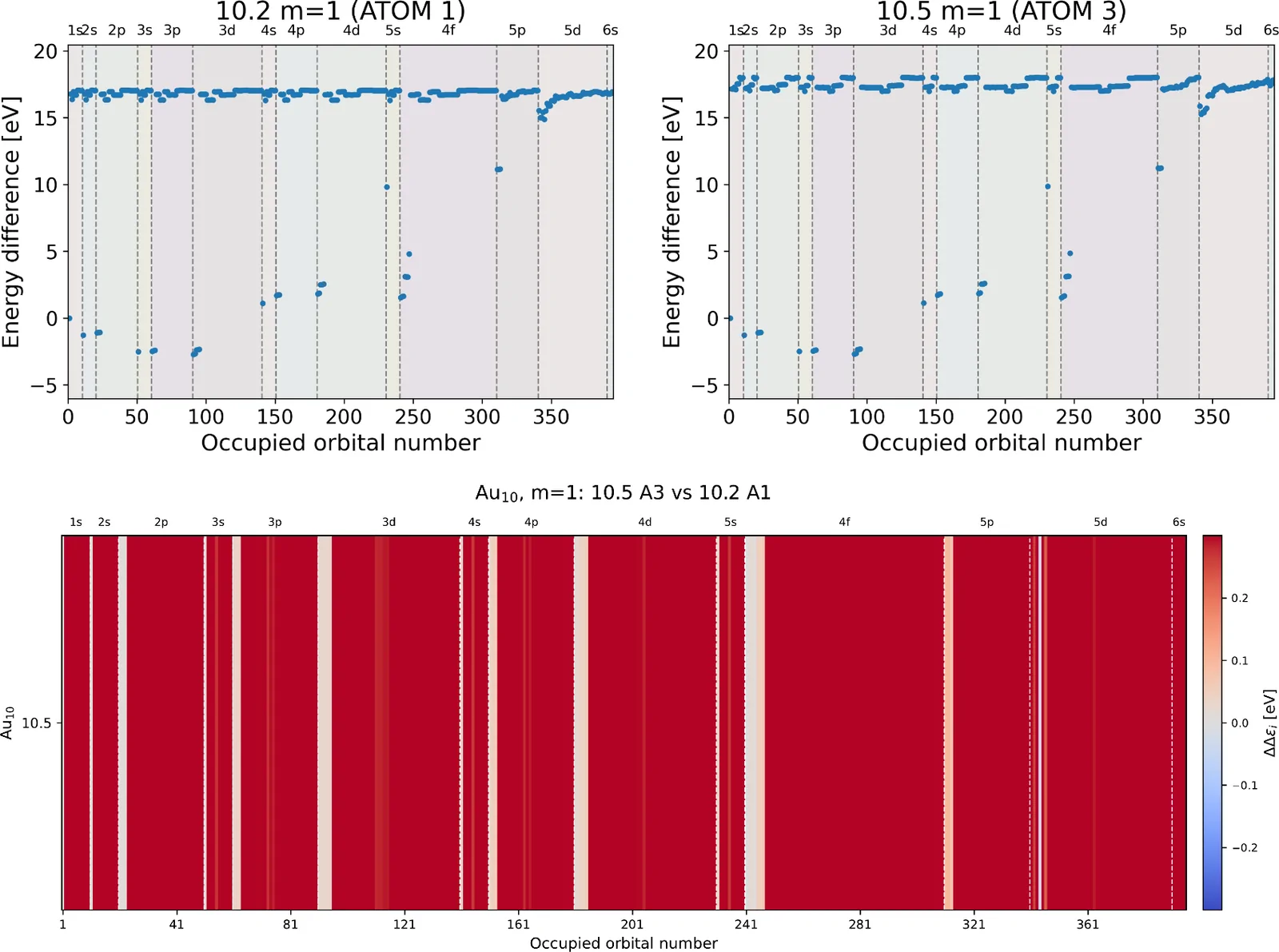
Δε_*i*_ patterns for selected ionized sites in Au_10_. The upper panels show the shifts for (**10.2**, A1, *m* = 1) and (**10.5**, A3, *m* = 1), while the heatmap shows the orbital-by-orbital difference of (**10.5**, A3, *m* = 1) relative to (**10.2**, A1, *m* = 1).

A similar trend is observed for atoms on which no explicit core–hole is imposed: their Kohn–Sham eigenvalues also undergo nearly rigid shifts, indicating that the nonionized atoms primarily track a cluster-wide change in the effective potential, whereas the genuinely local orbital-resolved reorganization remains concentrated on the hole-hosting atom. The Δε_*i*_ structure visible for Au_20_ (cf. Figure 10) shows that the occupied orbitals still retain a clear shell-like ordering with adjacent blocks maintaining predominantly atomic-subshell character despite bonding and delocalization. This further suggests that, as the cluster grows, an increasing fraction of the electronic structure participates in a more nearly rigid, metallic-like screening response, while the innermost core remains comparatively atomic-like.

**Figure 10:**
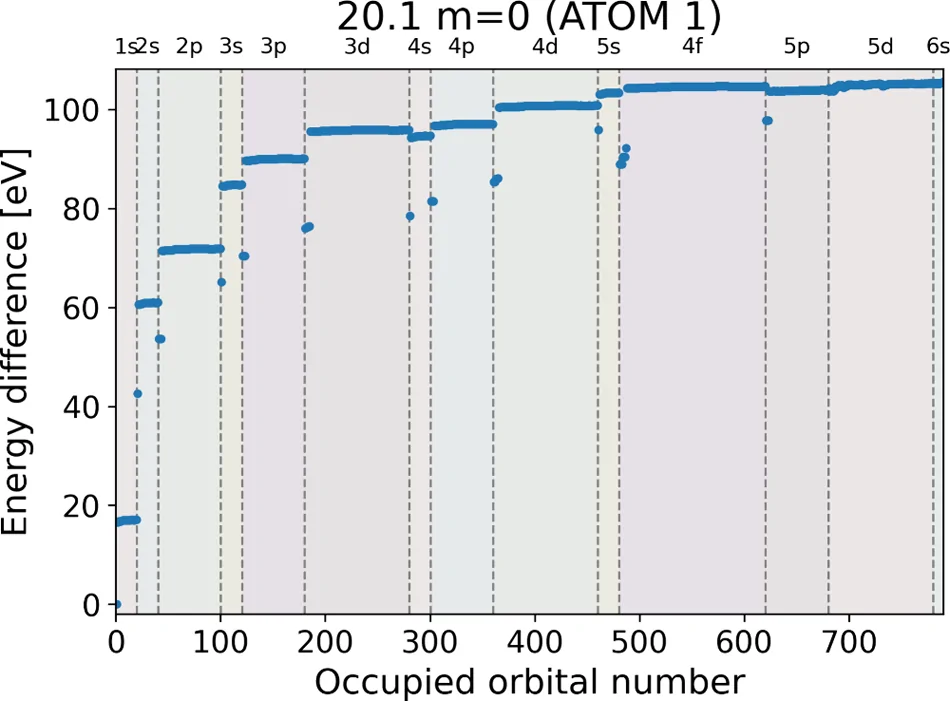
Δε_*i*_ as a function of orbital index for Au_20_ (*m* = 0). Colors indicate the dominant subshell character of the occupied states, which remain arranged in a clear shell-like sequence akin to atomic orbital ordering.

#### 4.2.7 Atom-Dependent Core-Level Shifts in the Au Clusters

Using the same median-based descriptor as for the TiO_2_ clusters, the CEBE shifts are shown to be governed primarily by site exposure but not by coordination number alone (cf. Table S12 in the Supporting Information). In the Au_4_ isomers, the lowest median CEBEs are consistently associated with the most weakly coordinated and most external sites: atom 1 in **4.1** and atom 2 in both **4.2** and **4.3**. In **4.2** and **4.3**, these minima correspond to 1-fold-coordinated atoms with *r*_norm_ = 1.00, whereas more embedded 3-fold-coordinated sites are shifted upward by about 0.36–0.49 eV.

A similar, but more nuanced, pattern persists in Au_10_. In **10.1**, the lowest CEBE is found for the most external 2-fold-coordinated site (atom 5), while atom 9, which is 3-fold coordinated but still relatively exposed, already lies 0.781 eV higher. In **10.2** and **10.4**, the lowest CEBEs are again associated with relatively external 3-fold-coordinated sites, whereas more embedded 6-fold-coordinated sites are shifted upward by 0.669 and 0.756 eV, respectively. However, **10.4** clearly shows that coordination alone is insufficient: atom 8 is only 2-fold coordinated and fully external yet still lies 0.190 eV above atom 3. The atom-dependent CEBE shifts in Au therefore reflect a combination of first-shell coordination and the more detailed local scaffold, including second-shell effects and the overall embedding of the ionized site within the cluster. The same trend continues in Au_20_, where the more densely embedded 9-fold-coordinated site lies 0.279 eV above the lower-binding-energy 6-fold-coordinated site.

Overall, both material classes show the same central feature: the screened core–hole state is characterized by a strongly stabilized but internally rather rigid inner core and by a more strongly reorganized outer shell. The local relaxation motif revealed by the Δε_*i*_ patterns is thus qualitatively similar in metallic Au clusters and in insulating TiO_2_ fragments, despite the very different electronic character of the two systems. This observation motivates the search for more compact descriptors of the full relaxation response, which is addressed in the following section. The corresponding CEBE shifts follow the same trend, indicating that the main differences between sites are controlled primarily by the efficiency of local screening. Accordingly, the absolute magnitude of the shifts may vary from system to system, but their physical origin remains the same.

### 4.3 Toward Compact Descriptors of CEBEs: Aggregated Eigenvalue Shifts (AESs)

The orbital-resolved eigenvalue shifts $\Delta \varepsilon_{i}^{k}$ introduced in eq 2 provide a detailed fingerprint of the core–hole response at a given site. For a more compact comparison between ΔSCF CEBEs and different *m*-resolved core–hole instantiations, it is nevertheless useful to merge the full set of shifts into a single scalar quantity.

To this end, I define the aggregated eigenvalue shift (AES) for each ionized calculation *k* as

5 $$\sum \Delta \varepsilon^{k}=\underset{i\in S}{\sum }\Delta \varepsilon_{i}^{k}$$

where *S* denotes the set of orbitals for which AES is defined. In the present work, *S* comprises all orbitals of the ionized atom that are occupied in the ground state. The quantities $\Delta \varepsilon_{i}^{k}$ are the aligned shifts discussed in Section 4.2. Within the first-order picture of eq 4, AES can be interpreted as an aggregate net response to the core–hole perturbation. All AES plots, including those discussed below, are available under 10.5281/zenodo.20067444.

#### 4.3.1 Observed Trends and Correlation Strengths

For each symmetry-inequivalent ionized site with at least 3 SCF-converged *m*-resolved instantiations, the ΔSCF CEBEs were plotted against the corresponding AES values.

For the Au clusters, where a 4f hole provides up to seven *m*-resolved realizations, the correlations are predominantly positive, often close to linear for a given atom in a given structure. Across the analyzed Au_4_, Au_10_, and Au_20_ data sets, the median Pearson coefficients *r* are approximately 0.97, 0.94, and 0.99, respectively. Thus, despite one case with a negative *r*, the Au data indicate that AES captures the dominant variation of the *m*-dependent response in a compact form. Representative examples are shown in Figure 11.

**Figure 11:**
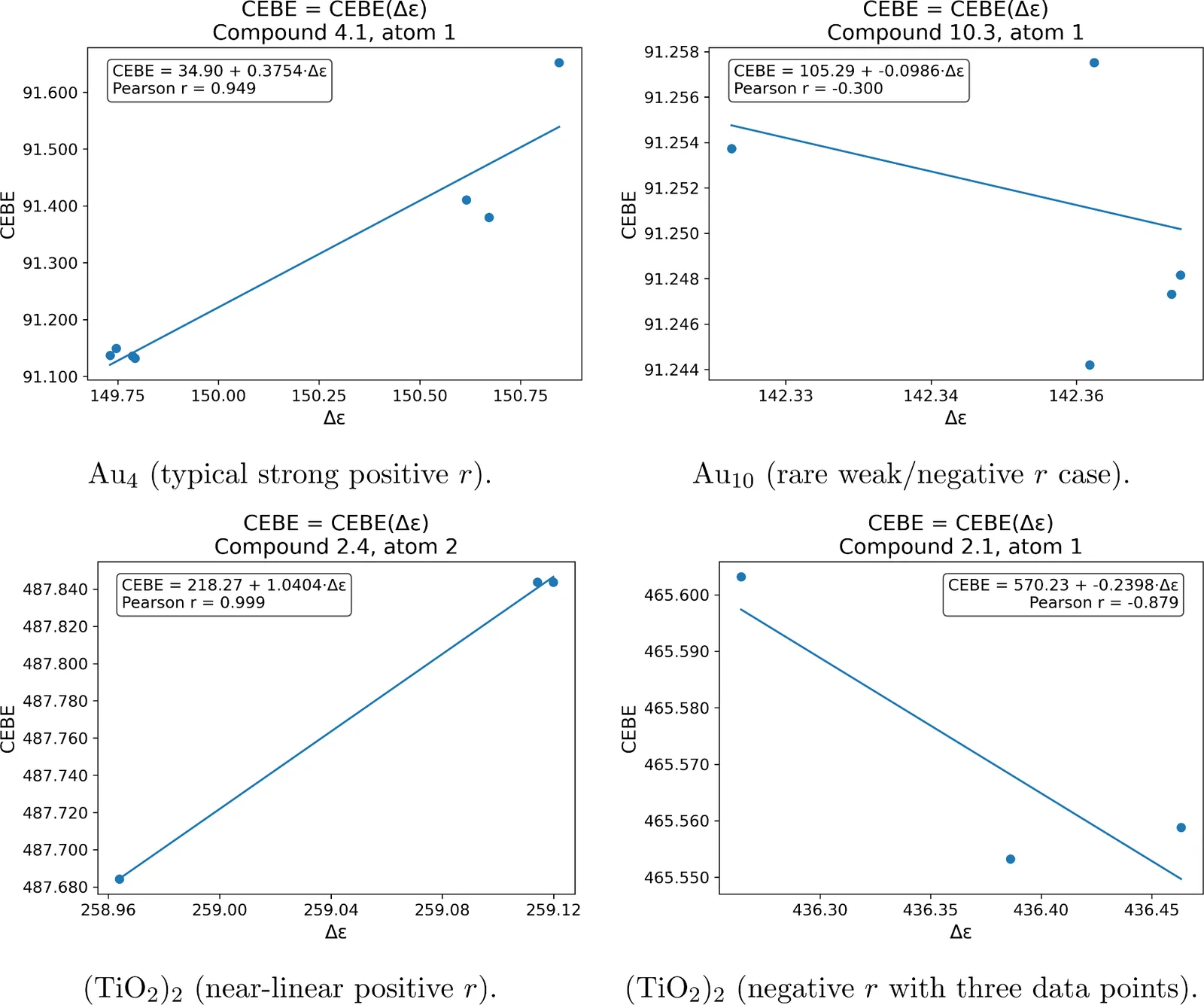
Representative correlations between ΔSCF CEBE and AES for different *m*-realizations of the same core–hole. Both CEBE and ΣΔε values (denoted here by Δε for typographic reasons) are given in eV.

For Ti 2p holes, the correlations are less definitive. Here, only 3 *m*-components are available, and the associated CEBE differences are usually very small. Pearson coefficients become thus highly sensitive to the minor differences, and both positive and negative values are obtained. The TiO_2_ results should therefore be viewed mainly as descriptive with their limitations discussed in the following.

#### 4.3.2 Origins of Nonpositive Pearson Correlations

It is important to note that low or nonpositive Pearson coefficients do not necessarily indicate a physically meaningful “anticorrelation” between CEBEs and AES. In the present data, two factors can account for such behavior.

1. *Small-n statistics and subtle CEBE range.* For a Ti 2p core–hole, only three *m* values are available. With *n* = 3, Pearson’s *r* is highly sensitive to minute differences in CEBEs, especially when the CEBE spread is only a few hundredths of an eV. In this regime, the sign of *r* should not be overinterpreted and nonpositive *r* values are better viewed just as a limitation of the scalar descriptor.
2. *Different physical observables.* CEBEs are total-energy differences, whereas ΣΔε is a compact descriptor derived from Kohn–Sham eigenvalues. The two quantities therefore need not vary one-to-one. In particular, AES combines subshell contributions that may shift in opposite directions, so a change dominated by valence-region reorganization need not track the corresponding total-energy response in a strictly monotonic way.

In summary, these points indicate that AES should be interpreted here only as an exploratory, proof-of-concept scalar summary of the full Δε_*i*_ response, not as a quantitatively robust standalone predictor of CEBEs. Even so, such a compact descriptor may already be useful for selected purposes: for example, for the Au clusters, where the *m*-dependent signal is larger and more systematic, AES still captures the dominant screening trend in a compact and physically interpretable way. For the TiO_2_ cases, however, the combination of sparse data and subtle CEBE variation prevents stronger quantitative claims.

## 5 Conclusions

In this work, I investigated site-resolved core–electron binding energies (CEBEs) and the orbital-resolved response to core ionization in two chemically distinct families of finite systems: metallic Au_*m*_ clusters (*m* = 4, 10, 20) and rutile- and anatase-precursor (TiO_2_)_*n*_ fragments (*n* = 2–4). Using a consistent ΔSCF protocol for core–hole final states within rSCAN DFT and a systematic treatment of symmetry-inequivalent sites, I quantified both cluster-to-cluster trends and intracluster site-to-site variability in computed CEBEs, i.e., the dispersion that contributes to inhomogeneous broadening in nanoscale XPS spectra. Beyond reporting absolute CEBEs, I analyzed the orbital-resolved Kohn–Sham eigenvalue shifts, Δε_*i*_, induced by a localized core–hole using a consistent internal alignment to the deepest 1s­(GS) level in order to relate them to the underlying relaxation and screening response in finite systems.

The ΔSCF results reveal different overall trends for the two cluster families. For (TiO_2_)_*n*_, the cluster-typical Ti 2p CEBEs remain essentially constant between *n* = 2 and *n* = 4, whereas the intracluster dispersion increases substantially with cluster size, reflecting the growing number of symmetry-inequivalent Ti sites and the increasing diversity of local Ti–O bonding patterns. Thus, for these oxide fragments, broadening from site heterogeneity is more important than any systematic centroid shift over the size range studied. In contrast, Au_*m*_ shows a statistically robust downshift of Au 4f CEBEs with increasing cluster size, consistent with the gradual buildup of metallic screening and delocalization as the clusters evolve toward more bulk-like behavior. This trend is confirmed by experimental data.

The revised structure-based analysis further shows that the site-dependent CEBEs can be interpreted directly in terms of the local environment of the ionized atom. For Au, the atom-dependent CEBE shifts are governed primarily by site exposure and local packing but not by coordination number alone: weakly coordinated, more external sites tend to exhibit lower median CEBEs, whereas more embedded sites are shifted to higher binding energy. For TiO_2_, the site dependence is more strongly chemical in character and cannot be reduced to the nominal Ti coordination number alone. Instead, the shifts depend on the detailed Ti–O connectivity, including the balance between terminal and bridging O ligands, the asymmetry of the local Ti–O environment, and the embedding of the ionized Ti site in the wider Ti–O–Ti scaffold. In this sense, the atom-dependent CEBEs provide a compact structural fingerprint complementary to the orbital-resolved Δε_*i*_ analysis.

Across both material classes, the orbital-resolved analysis uncovers a robust local motif of core–hole relaxation. Once the eigenvalue scale is referenced consistently, the Δε_*i*_ spectrum on the hole-hosting atom is characterized by a comparatively rigid inner-core region and by a much more strongly reorganized outer-shell region that carries most of the screening response. This “rigid inner core/flexible outer shell” structure appears largely universal for the systems studied, despite their very different bonding and electronic character. At the same time, the valence-region shifts remain sensitive to the chemical environment and connectivity, highlighting that the outer-shell part of Δε_*i*_ contains information on how a given site is embedded in the cluster and what screening pathways are available.

These results also underline that the interpretation of CEBEs in finite (sub)­nanoscale systems cannot be reduced only to elemental identity or to a simple structural classification of the ionized site. The core–hole response is governed by self-consistent many-electron relaxation and screening in a discrete level structure, where finite-size quantum effects, hybridization, and exchange–correlation contributions affect both the centroid and the distribution of binding energies. Consequently, neither a purely classical electrostatic picture nor continuum screening models are sufficient to rationalize site-specific trends across different cluster sizes and bonding motifs. Reliable modeling of such systems therefore requires a treatment that connects the local atomic environment with the corresponding electronic relaxation response, rather than relying only on geometric or classical chemical-environment arguments.

Finally, I examined whether the orbital-resolved relaxation fingerprints can be compressed into a simple scalar descriptor, such as the aggregated eigenvalue shift ΣΔε (AES). The results show that AESs should be regarded as proof-of-concept descriptors of the full Δε_*i*_ response, rather than as a quantitatively robust predictor of CEBEs. For the Au clusters, where the *m*-dependent signal is larger and more systematic, the AES captures the dominant trend in a compact way and may serve as a useful auxiliary descriptor for future data-driven screening or machine-learning workflows. For Ti 2p holes, however, the combination of sparse *m* sampling and very small CEBE spreads makes stronger quantitative claims impossible. In this regime, the orbital-resolved Δε_*i*_ patterns remain the physically more informative level of description.

In summary, the present study demonstrates that combining ΔSCF CEBEs with orbital-resolved eigenvalue shifts provides a unified electronic picture of finite-system core–hole screening and clarifies how this response depends on both cluster size and its local structure. In Au clusters, quantum-size effects manifest primarily as systematic centroid shifts together with site-dependent variations governed by exposure and packing, whereas in (TiO_2_)_*n*_ (*n* = 2–4), the centroid remains essentially unchanged and local chemical/topological heterogeneity dominates the spectral variability. Extending this framework to larger clusters and surfaces, incorporating a more complete treatment of relativistic effects for Au, and analyzing the real-space redistribution of screening charge would be natural next steps toward predictive, high-throughput modeling of XPS in complex nanostructures.

## Supplementary Material



## Notes

Tables S1–S8: calculated CEBE values for the studied TiO_2_ and Au clusters for the different *m* states at the symmetry-inequivalent atomic sites; Tables S9–S10: Mulliken Δ*q* values for selected core-ionized TiO_2_ clusters for different *m* indices; Tables S11–S12: representative atom-dependent CEBEs and local-environment descriptors for selected TiO_2_ and Au clusters (PDF)
