# Supplementary material for: Bridging Electron Density Relaxation and XPS Spectroscopy: Orbital-Resolved Trends in Core-Ionized Au m (m = 4, 10, 20) and (TiO2) n (n = 2–4) Clusters
Source: J Phys Chem A. 2026 Aug 28;130(36):7071–85. doi: 10.1021/acs.jpca.6c01107 (PMC13573611; doi:10.1021/acs.jpca.6c01107)
Supplement: Supplementary file 1 [file jp6c01107_si_001.pdf]

Bridging Electron Density Relaxation and XPS Spectroscopy: Orbital-Resolved Trends in Core-Ionized Au_m (m = 4, 10, 20) and $(TiO_2)_n$ (n = 2–4) Clusters

Supporting Information

Bartłomiej Gostyński*,†,‡

†*Department of Biophysics, Institute of Materials Science and Engineering*

ul. Stefanowskiego 1/15 90-537 Łódź, Poland

‡*Centre of Molecular and Macromolecular Studies Polish Academy of Sciences*

ul. Sienkiewicza 112 91-363 Łódź, Poland

E-mail: bartlomiej.gostynski@cbmm.lodz.pl

S1. Calculated geometries of the compounds

Any lack of congruence in the structure numbering, as for example **2.4** directly following **2.2** in the TiO_2 series, is directly attributable to the lack of convergent structures or their convergence to one of the other conformers (as in the case of compound **2.3**).

S1.1 TiO_2 clusters

1. Series $\mathbf{(TiO_2)_2}$

```
6
Compound TiO2 2.1
Ti 10.50797776  10.36259145  9.10198099
Ti 12.93485626  10.36256688 10.27853450
O  14.31151380  10.36261786  9.41680792
O  11.72142002  11.60950022  9.69025293
O  11.72146885   9.11565539  9.69015570
O   9.13149632  10.36255120  9.96398097
```

```
6
Compound TiO2 2.2
Ti   10.50797776  10.36259145   9.10198099
Ti   12.93485626  10.36256688  10.27853450
O    14.31151380  10.36261786   9.41680792
O    11.72142002  11.60950022   9.69025293
O    11.72146885   9.11565539   9.69015570
O     9.13149632  10.36255120   9.96398097
```

```
6
Compound TiO2 2.4
Ti    9.54879861  10.32737347  10.30942559
Ti   11.92843086  10.32797454  10.30916682
O    10.56270882  11.75156671  10.53720734
O    13.55630952  10.32789538  10.30913293
O    10.56375407   9.41834851  11.42844230
O    10.56313560   9.81307738   8.96224504
```

2. Series $\mathbf{(TiO_2)_3}$

```
9
```

Compound TiO2 3.1

Ti 11.84148964 12.56430457 10.58938145

Ti 13.25746524 10.24000230   9.99215580

Ti 10.42542233 10.24005016   9.99218960

O   13.24801169 12.20776965   9.65024678

O   10.43493664 12.20781749   9.65027673

O   14.54970578   9.42927550 10.54236911

O   9.13317149   9.42936835 10.54244800

O 11.841418000   9.33515257   9.21880567

O 11.841470870 10.92347186 11.32812836

9

Compound TiO2 3.2

Ti 12.93120843 10.20856439 10.17185998

Ti 15.59314404 10.28046737 10.59781843

Ti 10.26930206 10.34085742   9.76170516

O   16.61945899   9.40647557   9.70166564

O   9.24313608   9.34890542 10.52540320

O   11.51971626 11.09779952 10.90073986

O   14.34371948 11.19139205   9.57695095

O   11.70656595   9.45918360   9.02917311

O   14.15490418   9.30442316 11.19714302

9

Compound TiO2 3.4

Ti 13.65544955 11.81737091 10.18771628

Ti 10.94152619 10.14993541   9.25955009

Ti 10.92603074 13.45950741   9.25983938

O   15.01322279 11.82502965   9.30390124

```
O    9.96624186   9.33177048  10.25866916
O    9.94183938  14.27027424  10.25625453
O   12.62893175  13.30729739   9.91326715
O   12.64386583  10.31774390   9.91056912
O   10.31184691  11.80184110   8.76141304
```

3. Series $(\mathbf{TiO_2})_4$

```
12
Compound TiO2 4.1
Ti  12.96996902  12.19643632  10.11680114
Ti  12.96995542   9.43100227  10.11678500
Ti  10.53567289  10.81373082  10.37011374
Ti  15.40419236  10.81371058   9.86308270
O   12.19613062  10.81372757   9.12193493
O   13.74387934  10.81371164  11.11157532
O   11.34812581  12.50822173  10.85046839
O   14.59168143   9.11921322   9.38286921
O   11.34811383   9.11922464  10.85045353
O   14.59169293  12.50822161   9.38288577
O    9.15597086  10.81374445   9.53497283
O   16.78402752  10.81370115  10.69799920
```

```
12
Compound TiO2 4.2
Ti  13.97534640  10.28894351  11.10746546
Ti   9.95323117  10.28887912  11.10741428
Ti  11.96432368  11.79428746   9.73333061
Ti  11.96428296  11.74138437  12.53736494
```

```
O   10.29447472  11.14554024  12.81252759
O   13.63414824  11.20932874   9.43585215
O   11.96428761  10.38408029  11.10922627
O   13.63410095  11.14560141  12.81258217
O   10.29452374  11.20925978   9.43582059
O   14.66555853   8.83241332  11.08005907
O    9.26305074   8.83233367  11.07998956
O   11.96427712  12.93741174  11.15743130

12
Compound TiO2 4.3
Ti  13.65745490  12.08469578  10.68038105
Ti  12.06057594   9.75582953   9.11618800
Ti  10.46363622  12.08469770  10.68031709
Ti  12.06051025   9.75622602  12.24509592
O   14.99616153  13.00303810  10.68029934
O   10.68128358  10.77089271   9.20130218
O   13.43986873  10.77088691   9.20135695
O   12.06054170   8.80961505  10.68076615
O   10.68121841  10.77126824  12.15966677
O   12.06054748  12.99822289  10.68023020
O   13.43980693  10.77126432  12.15972243
O    9.12492846  13.00303873  10.68017853

12
Compound TiO2 4.5
Ti  10.75144627  10.83440613  10.35428893
Ti  13.58598050   9.10748399  10.35431257
Ti  12.10758544  13.19128571  10.35433051
```

Ti 14.9421196 11.46436452 10.35429061

O 11.83071220 9.25591364 10.35440599

O 9.14192695 10.64260779 10.35415013

O 14.27451835 10.11243261 9.11371297

O 11.41887313 12.18640474 11.59489211

O 11.41904524 12.18635176 9.11372180

O 16.55163847 11.65616425 10.35414570

O 13.86285401 13.04285731 10.35442032

O 14.27469585 10.11235016 11.59488309

12

Compound TiO2 4.6

Ti 13.27957223 9.98247845 10.66696048

Ti 12.34642723 12.40601180 9.77037095

Ti 15.03810240 12.52423780 11.48194984

Ti 9.96507614 11.59999535 10.93833451

O 13.08213136 11.00077291 9.02078921

O 14.72319114 10.73959936 11.57669208

O 11.79104763 11.20174647 11.21544243

O 13.59337273 13.37035930 10.61917447

O 9.29118071 12.36908849 12.18377692

O 10.52082681 12.73742283 9.63789222

O 12.93525060 8.39296340 10.74235888

O 16.47410954 12.87625983 10.83141746

12

Compound TiO2 4.7

Ti 14.87914301 11.08119096 9.52285757

Ti 10.25141936 11.69799502 10.25082129

Ti	13.22649698	13.35285653	10.27265459
Ti	12.94074230	10.05584730	11.28674178
O	13.70122785	9.48244350	9.86477250
O	16.45144671	10.88765058	9.86731468
O	14.36869440	12.72090350	8.93343002
O	13.78184158	11.62043459	11.24255021
O	11.20570533	10.28220394	11.12062782
O	9.54487871	11.27925775	8.85967392
O	13.65289560	14.72851167	11.02019880
O	11.36222016	13.06720867	10.06896042

12

Compound TiO2 4.8

Ti	10.45736022	9.68240483	10.35549823
Ti	13.75802989	12.42742430	10.35547841
Ti	13.73986694	9.68277143	10.35548475
Ti	10.43853149	12.42705236	10.35547566
O	13.84150313	10.85193043	9.11179737
O	10.35547075	10.85155126	11.59917554
O	12.09819000	13.21650613	10.35546022
O	12.09870598	8.87093506	10.35548130
O	13.84148896	10.85193563	11.59916774
O	10.35542777	10.85153011	9.11180387
O	15.05930550	13.39971834	10.35548775
O	9.13702698	13.39904411	10.35549444

12

Compound TiO2 4.10

Ti	12.45034467	13.86389410	10.67022272

Ti	14.31636639	12.09864729	9.56002978
Ti	12.44275333	10.34150194	10.67041204
Ti	9.56520061	12.10887897	10.57415169
O	12.98615289	15.08709743	11.58463965
O	13.40860117	12.10064943	11.12362754
O	12.97332355	9.11612536	11.58497347
O	10.63507373	10.63466666	10.72204150
O	13.53197475	13.53998097	9.03695942
O	13.52578851	10.66063070	9.03713636
O	8.65848823	12.11078063	9.23241874
O	10.64142616	13.57847452	10.72190911

12

Compound TiO2 4.11

Ti	12.37899942	9.64852841	9.87945548
Ti	13.18875808	14.15202737	10.64740912
Ti	15.28559974	11.35593824	10.16500320
Ti	10.75044799	11.74746759	10.51714930
O	14.55299909	12.90577855	10.73238975
O	11.55718663	13.39923056	10.78703484
O	11.81132451	10.30171368	11.36708652
O	13.33348620	15.07869461	9.32453989
O	14.13055945	9.96441852	9.68445356
O	9.15762318	11.63716826	10.80478378
O	16.59521673	10.90341855	10.99688346
O	11.42403438	10.74144040	8.94551108

12

Compound TiO2 4.12

```
Ti  10.24644845  12.09874316  11.04213992
Ti  12.67905912  12.55684624   8.90181962
Ti  12.67988507   9.86228804  10.66416530
Ti  15.11073406  12.10145008  11.04325749
O   11.11212515  10.59458922  11.43203316
O   14.14887186  13.09270988   9.71428918
O   11.20817958  13.09078762   9.71368113
O   12.68023413  10.84006967   8.93010554
O   12.68132173   8.25036706  10.47292010
O   14.24567999  10.59706981  11.43371519
O   15.57850734  12.93811394  12.34432127
O    9.77899952  12.93486528  12.34364345
```

S1.2 Au clusters

1. Series $(\mathbf{Au})_4$

```
4
Compound Au 4.1
Au  3.94865712   3.56614249  -1.01361106
Au  3.37831448   1.15069162  -0.07715416
Au  1.55402907  -0.38896897  -1.23300351
Au  2.12399934   2.02613487  -2.16923127

4
Compound Au 4.2
Au   4.01027979  2.31385880  -1.66564200
Au  -0.79822605  3.59842422  -1.77770331
Au   2.74401187  0.09608810  -1.75062670
```

Au 1.3609344 2.35762889 -1.74202799

4

Compound Au 4.3

Au 3.99357772 2.20911018 -1.66111958

Au -0.03454571 4.49489224 -1.71304321

Au 5.48146916 0.21038178 -1.66054157

Au 1.46849883 2.50761579 -1.73829564

2. Series $\mathbf{(Au)_{10}}$

10

Compound Au 10.1

Au 0.00000034 1.53460264 -1.70172235

Au -1.32930805 -0.76780884 -1.70197289

Au -1.39142508 -0.80335169 1.01936840

Au -0.00000038 1.60656869 1.01963071

Au -3.29520191 -1.90133183 -0.36496422

Au -0.00000002 3.80406576 -0.36509528

Au 1.39142464 -0.80335149 1.01936777

Au 1.32930840 -0.76780901 -1.70197350

Au 0.00000016 -0.00025235 3.14232132

Au 3.29520190 -1.90133186 -0.36496416

10

Compound Au 10.2

Au 0.00000005 2.35435713 0.00000011

Au 0.00000002 -2.35435710 0.00000003

Au 0.00000070 -2.33043790 2.65435399

Au -0.00000017 0.00000002 4.00359609

Au 0.00000005 2.33043783 2.65435395

Au 0.00000003 0.00000005 1.34594138

Au 0.00000004 -0.00000011 -1.34594132

Au 0.00000004 2.33043797 -2.65435407

Au 0.00000004 -2.33043787 -2.65435400

Au -0.00000016 -0.00000002 -4.00359617

10

Compound Au 10.3

Au -1.95098768 -0.07487034 -0.36237610

Au -0.86699336 -0.95370599 2.01011379

Au 0.86699336 0.95370595 2.01011376

Au -0.12081025 1.94222370 -0.35926945

Au -2.36008483 2.13708707 -1.66056230

Au 1.95098767 0.07487036 -0.36237616

Au 0.12081025 -1.94222368 -0.35926945

Au -2.35579396 -2.60369218 0.37209408

Au 2.35579395 2.60369217 0.37209410

Au 2.36008483 -2.13708709 -1.66056227

10

Compound Au 10.4

Au -0.00000011 2.64429968 -1.42473940

Au -0.00000024 -2.64429967 -1.42473940

Au 0.00000017 -1.32150095 -3.73696029

Au 0.00000020 1.32150095 -3.73696028

Au -0.00000014 1.42491943 0.92564828

Au -0.00000010 -0.00000001 -1.35994197

Au	-0.00000009	-1.42491943	0.92564827
Au	0.00000015	-2.57537447	3.28341460
Au	0.00000011	0.00000000	3.26521459
Au	0.00000004	2.57537447	3.28341460

10

Compound Au 10.5

Au	0.00000000	2.59134715	3.27670430
Au	-0.00000017	1.35640750	0.97253567
Au	0.00000007	0.00000002	3.40385045
Au	-1.35640763	-0.00000002	-0.97253515
Au	0.00000015	-2.59134708	3.27670452
Au	-0.00000003	-1.35640767	0.97253577
Au	1.35640755	-0.00000009	-0.97253511
Au	-2.59134707	0.00000028	-3.27670387
Au	2.59134707	-0.00000002	-3.27670373
Au	0.00000005	0.00000010	-3.40384985

10

Compound Au 10.6

Au	0.00000002	0.00000000	3.32952040
Au	-2.17649307	0.00000015	1.62767621
Au	-1.52233906	-0.00000008	-0.96112381
Au	0.00000009	1.52233906	0.96112381
Au	-0.00000009	-1.52233907	0.96112379
Au	1.52233902	0.00000010	-0.96112386
Au	2.17649308	-0.00000014	1.62767618
Au	0.00000009	-2.17649304	-1.62767616
Au	-0.00000021	2.17649301	-1.62767619

```
Au   0.00000013   0.00000002  -3.32952036

10
Compound Au 10.7
Au   1.59853212   0.92310967   2.68133177
Au   4.12801038   2.38319013  -1.68521453
Au   1.12071955  -0.89151010   0.63043810
Au   2.83814436   1.63869497   0.54673539
Au   1.59854048  -2.22064447  -1.76412594
Au  -0.21159272   1.41630764   0.63039372
Au  -0.21148522  -0.12217737  -1.54545757
Au   2.83818030   0.03095058  -1.72720311
Au  -1.12365454   2.49456554  -1.76381557
Au   1.44585608   2.44268631  -1.72723394
```

3. Series $\mathbf{(Au)_{20}}$

```
20
Compound Au 20.1
Au   1.45220537   0.83849414   2.80462654
Au   3.99130880   2.30437890  -1.62948816
Au  -1.56650777  -0.90444927   0.63957095
Au   1.56650777  -0.90444927   0.63957094
Au  -1.45220540   0.83849415   2.80462645
Au   0.00000001  -3.20325970   0.64607944
Au   2.77409151   1.60162150   0.64630427
Au   1.45214809  -2.36488755  -1.72543568
Au   0.00000000  -1.67689700   2.80465261
Au  -1.45214807  -2.36488757  -1.72543568
```

Au	-0.00000007	1.80885588	0.63961478
Au	-2.77409143	1.60162145	0.64630433
Au	0.00000000	-0.00009346	-1.91841504
Au	-0.00000001	-4.60862637	-1.62945967
Au	0.00000002	0.00000255	4.88814624
Au	-2.77419326	-0.07534856	-1.72541678
Au	2.77419324	-0.07534858	-1.72541678
Au	-3.99130885	2.30437893	-1.62948819
Au	-1.32194663	2.44018843	-1.72547029
Au	1.32194667	2.44018840	-1.72547030

S2. Calculated CEBE values

1. Series $(\mathbf{TiO_2})_2$

Table S1: CEBE values for the TiO_2 **2.x** series in the three distinct m states at the symmetry inequivalent atoms. Numbers of the symmetry-inequivalent atoms correspond to the atom numbers given in the S1 section.

**2.1**	$m = -1$	$m = 0$	$m = 1$
Atom 1	465.60	465.56	465.55
**2.2**	$m = -1$	$m = 0$	$m = 1$
Atom 1	465.63	465.54	465.54
**2.4**	$m = -1$	$m = 0$	$m = 1$
Atom 1	466.29	466.28	466.31
Atom 2	487.84	487.84	487.68

2. Series $(\mathbf{TiO_2})_3$

Table S2: CEBE values for the TiO_2 **3.x** series in the three distinct m states at the symmetry inequivalent atoms. Numbers of the symmetry-inequivalent atoms correspond to the atom numbers given in the S1 section. N/A denotes when the density did not converge.

**3.1**	$m = -1$	$m = 0$	$m = 1$
Atom 1	466.64	N/A	466.89
Atom 2	465.66	465.66	465.54
**3.2**	$m = -1$	$m = 0$	$m = 1$
Atom 1	N/A	465.73	465.73
Atom 2	N/A	465.62	465.57
**3.4**	$m = -1$	$m = 0$	$m = 1$
Atom 1	465.63	465.57	465.57
Atom 2	N/A	N/A	N/A

3. Series $\mathbf{(TiO_2)_4}$

Table S3: CEBE values for the TiO_2 **4.x** series in the three distinct m states at the symmetry inequivalent atoms. Numbers of the symmetry-inequivalent atoms correspond to the atom numbers given in the S1 section. N/A denotes when the density did not converge.

**4.1**	$m=-1$	$m=0$	$m=1$
Atom 1	466.88	466.91	466.87
Atom 3	465.90	465.89	465.85
**4.2**			
Atom 1	465.89	466.01	N/A
Atom 3	466.73	466.70	466.66
**4.3**			
Atom 1	465.00	464.99	464.84
Atom 2	466.07	N/A	N/A
**4.5**			
Atom 1	465.25	465.22	465.16
Atom 2	465.97	466.10	466.08
**4.6**			
Atom 1	465.32	465.44	465.44
Atom 2	N/A	466.29	N/A
Atom 3	465.62	N/A	465.62
Atom 4	N/A	466.26	N/A
**4.7**			
Atom 1	465.42	465.41	465.37
Atom 2	N/A	N/A	N/A
Atom 3	464.97	465.01609	643.78
Atom 4	N/A	N/A	N/A
**4.8**			
Atom 1	N/A	466.16	465.98
Atom 2	465.00	464.97	464.88
**4.10**			
Atom 1	465.47	N/A	N/A
Atom 2	466.49	N/A	466.49
Atom 4	465.34	465.30	465.30

Table S4: continued: CEBE values for the TiO_2 **4.x** series in the three distinct m states at the symmetry inequivalent atoms. Numbers of the symmetry-inequivalent atoms correspond to the atom numbers given in the S1 section. N/A denotes when the density did not converge.

**4.11**	$m = -1$	$m = 0$	$m = 1$
Atom 1	466.05	N/A	N/A
Atom 2	N/A	N/A	N/A
Atom 3	465.72	465.64	N/A
Atom 4	N/A	465.06	465.00
**4.12**			
Atom 1	N/A	N/A	N/A
Atom 2	N/A	466.37	466.51
Atom 3	464.78	464.88	464.89

4. Series **(Au)$_4$**

Table S5: CEBE values for the Au **4.x** series in the seven distinct m states at the symmetry inequivalent atoms. Numbers of the symmetry-inequivalent atoms correspond to the atom numbers given in the S1 section.

**4.1**	$m=-3$	$m=-2$	$m=-1$	$m=0$	$m=1$	$m=2$	$m=3$
Atom 1	91.41	91.15	91.14	91.14	91.65	91.13	91.38
Atom 2	92.04	91.81	91.81	91.82	91.81	91.81	92.03
**4.2**							
Atom 1	92.06	91.83	91.82	92.11	91.81	91.83	92.33
Atom 2	90.88	90.64	91.14	90.90	90.63	90.62	90.88
Atom 4	91.58	91.37	91.90	91.36	91.36	91.35	91.62
**4.4**							
Atom 1	91.78	91.54	91.56	91.84	91.56	91.56	91.81
Atom 2	91.72	91.18	91.20	91.47	91.19	91.20	91.47

4. Series $\mathbf{(Au)_{10}}$

Table S6: CEBE values for the Au **10.x** series in the seven distinct m states at the symmetry inequivalent atoms. Numbers of the symmetry-inequivalent atoms correspond to the atom numbers given in the S1 section. N/A denotes when the density did not converge.

**10.1**	$m=-3$	$m=-2$	$m=-1$	$m=0$	$m=1$	$m=2$	$m=3$
Atom 1	91.21	90.95	90.97	90.97	91.53	90.97	91.25
Atom 3	91.59	91.36	91.36	91.86	91.84	91.35	91.36
Atom 5	91.17	90.94	90.92	91.20	90.93	90.92	91.20
Atom 9	92.24	91.71	91.72	91.72	91.71	91.72	92.24
**10.2**							
Atom 1	91.51	91.22	91.24	91.48	91.24	91.24	91.53
Atom 3	91.49	90.98	91.21	91.47	90.96	90.98	91.26
Atom 4	91.15	90.88	90.90	91.17	91.44	90.90	91.19
Atom 6	91.82	91.55	92.04	91.83	91.57	91.57	91.88
**10.3**							
Atom 1	91.26	91.24	91.25	91.26	91.25	91.25	N/A
Atom 2	N/A	90.84	91.35	90.83	90.83	90.84	91.12
Atom 5	91.57	91.05	91.34	91.57	91.59	91.06	91.34
Atom 8	92.04	91.81	91.81	91.82	91.81	91.81	92.03
**10.4**							
Atom 1	91.62	91.07	91.32	91.09	91.08	91.08	91.38
Atom 3	91.13	90.84	91.33	90.84	90.83	90.84	91.12
Atom 5	91.16	91.16	91.16	91.16	91.15	91.16	91.46
Atom 6	91.85	91.58	91.60	91.85	91.59	91.60	91.89
Atom 8	91.55	91.01	91.03	91.54	91.02	91.02	91.31
Atom 9	91.41	91.15	91.17	91.45	91.17	91.18	91.47
**10.5**							
Atom 1	91.40	91.11	91.37	91.64	91.66	91.37	91.40
Atom 2	91.49	91.21	91.23	91.23	91.74	91.22	91.52
Atom 3	91.31	91.05	91.07	91.35	91.07	91.08	91.37

Table S7: continued: CEBE values for the Au **10.x** series in the seven distinct m states at the symmetry inequivalent atoms. Numbers of the symmetry-inequivalent atoms correspond to the atom numbers given in the S1 section. N/A denotes when the density did not converge.

**10.6**	$m=-3$	$m=-2$	$m=-1$	$m=0$	$m=1$	$m=2$	$m=3$
Atom 1	91.16	90.86	90.88	91.14	90.87	90.88	91.14
Atom 2	91.47	91.17	91.18	91.19	91.41	91.18	91.72
Atom 3	91.58	91.34	91.33	91.34	91.56	91.34	91.63
**10.7**							
Atom 1	91.47	90.95	90.94	90.95	90.96	90.95	91.21
Atom 2	N/A	91.24	91.24	91.25	91.25	91.24	N/A
Atom 3	90.95	90.94	90.95	90.95	91.47	90.95	91.24
Atom 4	91.19	91.18	91.20	91.43	91.19	91.19	91.46

4. Series **$(Au)_{20}$**

Table S8: CEBE values for the Au **20.1** species in the seven distinct m states at the symmetry inequivalent atoms. Numbers of the symmetry-inequivalent atoms correspond to the atom numbers given in the S1 section.

**20.1**	$m=-3$	$m=-2$	$m=-1$	$m=0$	$m=1$	$m=2$	$m=3$
Atom 1	91.23	90.98	90.97	90.98	91.23	90.98	91.23
Atom 2	91.36	91.08	91.08	91.09	91.08	91.08	91.60
Atom 3	N/A	91.17	91.35	91.40	90.91	90.92	91.44

Table S9: Mulliken Δq Values for different m index for compounds **2.1 - 2.4**. Values for the ionized atoms are marked with bold font.

	m				m		
Atom nr	-1	0	1	Atom nr	-1	0	1
**2.1**				**2.2**			
1	**0.180**	**0.138**	**0.116**	1	**0.172**	**0.104**	**0.105**
2	-0.245	-0.246	-0.245	2	-0.241	-0.243	-0.243
3	-0.152	-0.147	-0.145	3	-0.142	-0.136	-0.136
4	-0.262	-0.253	-0.249	4	-0.255	-0.224	-0.225
5	-0.262	-0.253	-0.249	5	-0.267	-0.251	-0.250
6	-0.258	-0.239	-0.228	6	-0.267	-0.251	-0.250
**2.4**				**2.4**			
1	**0.336**	**0.336**	**0.332**	1	-0.231	-0.231	-0.251
2	-0.353	-0.354	-0.356	2	**0.597**	**0.597**	**0.551**
3	-0.268	-0.267	-0.266	3	-0.354	-0.346	-0.336
4	-0.176	-0.177	-0.179	4	-0.316	-0.316	-0.293
5	-0.271	-0.270	-0.266	5	-0.347	-0.353	-0.336
6	-0.268	-0.268	-0.266	6	-0.349	-0.351	-0.336

Table S10: Mulliken Δq values for different m index for Ti1(**3.2**), Ti2(**3.2**) and Ti1(**3.4**). Values for the ionized atoms are marked with bold font. Instances exhibiting electron density divergence are marked with N/A.

	m				m		
Atom nr	−1	0	1	Atom nr	−1	0	1
Ti1(**3.2**)				Ti2(**3.2**)			
1	**N/A**	**0.503**	**0.482**	1	N/A	−0.183	−0.185
2	N/A	−0.199	−0.210	2	N/A	**0.254**	**0.183**
3	N/A	−0.199	−0.210	3	N/A	−0.104	−0.092
4	N/A	−0.119	−0.117	4	N/A	−0.234	−0.211
5	N/A	−0.119	−0.117	5	N/A	−0.102	−0.084
6	N/A	−0.218	−0.208	6	N/A	−0.071	−0.068
7	N/A	−0.219	−0.208	7	N/A	−0.240	−0.236
8	N/A	−0.214	−0.206	8	N/A	−0.066	−0.060
9	N/A	−0.216	−0.206	9	N/A	−0.254	−0.247

Ti1(**3.4**)			
Atom nr	−1	0	1
1	**0.323**	**0.319**	**0.251**
2	−0.121	-0.122	−0.114
3	−0.121	-0.122	−0.114
4	−0.235	-0.238	−0.212
5	−0.114	-0.116	−0.106
6	−0.114	-0.116	−0.106
7	−0.282	-0.275	−0.273
8	−0.282	-0.275	−0.273
9	−0.054	-0.054	−0.054

S3. Calculated representative CEBE shifts and other descriptors

Table S11: Representative atom-dependent CEBEs for selected Ti sites in TiO_2 clusters. N_O is the number of O neighbors within Ti–O cutoff (2.275 Å), N_{term} and N_{bridg} are the numbers of terminal and bridging O ligands, respectively, and $\langle d_{Ti-O} \rangle / \Delta d_{Ti-O}$ lists the mean Ti–O distance and the Ti–O asymmetry span ($d_{max} - d_{min}$).

Cluster / isomer	Atom	$\widetilde{\text{CEBE}}$ (eV)	$\Delta\widetilde{\text{CEBE}}$ (eV)	N_O	N_{term}	N_{bridg}	$\langle d_{Ti-O} \rangle / \Delta d_{Ti-O}$ (Å)
$(TiO_2)_2$ / 2.4	1	466.288	0.000	3	0	3	1.763 / 0.000
$(TiO_2)_2$ / 2.4	2	487.844	21.556	4	1	3	1.896 / 0.358
$(TiO_2)_3$ / 3.2	2	465.595	0.000	3	1	2	1.770 / 0.234
$(TiO_2)_3$ / 3.2	1	465.728	0.133	4	0	4	1.828 / 0.014
$(TiO_2)_3$ / 3.4	1	465.570	0.000	3	1	2	1.760 / 0.210
$(TiO_2)_4$ / 4.10	4	465.301	0.000	3	1	2	1.758 / 0.208
$(TiO_2)_4$ / 4.10	2	466.490	1.189	3	0	2	1.751 / 0.086
$(TiO_2)_4$ / 4.12	3	464.876	0.000	4	1	3	1.850 / 0.368
$(TiO_2)_4$ / 4.12	2	466.439	1.563	3	0	3	1.748 / 0.046

Table S12: Representative atom-dependent CEBEs for selected Au clusters. N_{Au} is the number of Au neighbors within the Au–Au cutoff (3.275 Å), $\langle d_{Au-Au} \rangle$ is the mean Au–Au distance within that local shell, and r_{norm} is the normalized radial distance from the cluster center.

Cluster / isomer	Atom	$\widetilde{\text{CEBE}}$ (eV)	$\Delta\widetilde{\text{CEBE}}$ (eV)	N_{Au}	$\langle d_{Au-Au} \rangle$ (Å)	r_{norm}
Au_4 / 4.1	1	91.149	0.000	2	2.65	1.00
Au_4 / 4.1	2	91.812	0.663	3	2.63	0.56
Au_4 / 4.2	2	90.875	0.000	1	2.49	1.00
Au_4 / 4.2	4	91.365	0.490	3	2.60	0.18
Au_4 / 4.3	2	91.199	0.000	1	2.49	1.00
Au_{10} / 10.1	5	90.940	0.000	2	2.62	1.00
Au_{10} / 10.1	9	91.721	0.781	3	2.66	0.82
Au_{10} / 10.2	4	91.155	0.000	3	2.68	1.00
Au_{10} / 10.2	6	91.824	0.669	6	2.69	0.34
Au_{10} / 10.4	3	90.841	0.000	3	2.68	0.95
Au_{10} / 10.4	8	91.031	0.190	2	2.60	1.00
Au_{10} / 10.4	6	91.597	0.756	6	2.69	0.33
Au_{20} / 20.1	1	90.982	0.000	6	2.81	0.67
Au_{20} / 20.1	3	91.261	0.279	9	2.92	0.39

Acknowledgements

This paper was funded by the Horizon Europe program, the European Health and Digital Executive Agency (HADEA), grant number 101137990 - Project CheMatSustain

We gratefully acknowledge Polish high-performance computing infrastructure PLGrid (HPC Center: ACK Cyfronet AGH) for providing computer facilities and support within computational grants no. PLG/2024/017615 and PLG/2025/018739
